# Prediction Models and Risk Factors for Steroid Resistance in Children with Nephrotic Syndrome: A Systematic Review and Meta-Analysis

**DOI:** 10.3390/jcm15124438

**Published:** 2026-06-08

**Authors:** Yuanhui Hu, Zehui Zhang, Sha Diao, Yannan Guo, Yangtingting Gao, Zheng Xu, Qilin Peng, Yao Xu, Zhenyan Bo, Linan Zeng, Liang Huang, Jingjing Chen, Yizhun Zhu, Hailong Li, Lingli Zhang

**Affiliations:** 1 Department of Pharmacy/Evidence-Based Pharmacy Center, West China Second University Hospital, Sichuan University, No. 20, Section 3, Renmin South Road, Wuhou District, Chengdu 610041, China; huyuanhui@stu.scu.edu.cn (Y.H.);; 2 Children’s Medicine Key Laboratory of Sichuan Province, No. 20, Section 3, Renmin South Road, Wuhou District, Chengdu 610041, China; 3 NMPA Key Laboratory for Technical Research on Drug Products In Vitro and In Vivo Correlation, No. 20, Section 3, Renmin South Road, Wuhou District, Chengdu 610041, China; 4 Key Laboratory of Birth Defects and Related Diseases of Women and Children, Sichuan University, Ministry of Education, No. 20, Section 3, Renmin South Road, Wuhou District, Chengdu 610041, China; 5 West China School of Pharmacy, Sichuan University, No. 17, Section 3, Renmin South Road, Wuhou District, Chengdu 610041, China; 6 Department of Pediatrics, West China Second University Hospital, Sichuan University, No. 20, Section 3, Renmin South Road, Wuhou District, Chengdu 610041, China; 7 West China School of Medicine, Sichuan University, No. 17, Section 3, Renmin South Road, Wuhou District, Chengdu 610041, China; 8 West China Biomedical Big Data Center, West China Hospital, Sichuan University, No. 37, Guoxue Lane, Wuhou District, Chengdu 610041, China; 9 School of Pharmacy, Macau University of Science and Technology, No. 100-460, Avenida Wai Long, Taipa, Macao 999078, China; 10 Chinese Evidence-Based Medicine Center, West China Hospital, Sichuan University, No. 37, Guoxue Lane, Wuhou District, Chengdu 610041, China

**Keywords:** nephrotic syndrome, prediction model, risk factor, steroid resistance

## Abstract

**Background:** Steroid resistance indicates poor prognosis in pediatric nephrotic syndrome, but predictive models and risk factors for steroid-resistant nephrotic syndrome (SRNS) remain poorly understood. **Methods:** We searched PubMed, Embase, Scopus, CNKI, SinoMed, Wanfang, and VIP (inception to 1 March 2025) for studies developing SRNS prediction models or identifying risk factors. Odds ratios and AUC were pooled using random-effects meta-analysis. Risk of bias was assessed with PROBAST and Newcastle-Ottawa Scale. **Results:** Out of 2264 studies, 23 were included. Prediction models were mainly developed using logistic regression (16/17, 94.1%). The most frequently reported predictors included erythrocyte sedimentation rate and vitamin D binding protein. The reported AUC ranged from 0.75 to 0.88. Only one model had undergone external validation with an accuracy of 0.94. A total of 22 independent risk factors were identified, five of which—low birth weight, decreased urine output, hypertension, serum albumin, and serum IgM—were not in existing models. In total, 76% of model studies and 26% of risk factor analyses were at high or moderate risk of bias. **Conclusions:** Existing SRNS prediction models reported apparent discrimination but had a high risk of bias and very limited external validation, which substantially restricts their current clinical applicability. Several relevant risk factors remain unincorporated. Future research should prioritize rigorous model development and multi-center external validation.

## 1. Introduction

Pediatric primary nephrotic syndrome (PNS) is a common glomerular disorder marked by severe proteinuria, low serum protein, edema, and elevated lipid levels, with a reported prevalence between 1.15 and 16.9 per 100,000 [1]. Standard treatment involves glucocorticoids, yet around 10–20% of patients respond poorly [1], resulting in a diagnosis of steroid-resistant nephrotic syndrome (SRNS).

The response of children with PNS to glucocorticoid therapy is a critical factor in determining prognosis. By 10 years after diagnosis, the estimated risk of progression to chronic kidney disease among patients with steroid-sensitive nephrotic syndrome (SSNS) is below 5% [2], whereas the 10-year renal survival without progression to end-stage kidney disease among patients with SRNS has been reported to range between 35% and 51% [3]. No evidence supports the efficacy of long-term glucocorticoid therapy for SRNS patients; however, it may cause cumulative steroid-related adverse effects [4], and approximately 70% of SRNS patients may face a high risk of disease progression due to long-term ineffective hormone therapy [5].

The prediction of glucocorticoid response in nephrotic syndrome can assist clinicians in identifying potential hormone resistance in children with PNS and guide the development of optimal treatment strategies in clinical practice, thus shortening the time to urine protein remission and reducing both the length of hospital stay and treatment costs [6]. Furthermore, the guidelines from the Kidney Disease: Improving Global Outcomes (KDIGO) organization recommend assessing patient prognosis based on the initial response to glucocorticoid therapy to minimize unnecessary renal biopsies [7].

Although various SRNS prediction models have been developed, these models vary in methodology, performance, and applicability, and no study has systematically compared model performance across studies applied to SRNS. Additionally, the predictors across studies vary significantly, resulting in uncertainty regarding their clinical applicability.

Systematic reviews have highlighted numerous biomarkers available for predicting SRNS [8,9]; however, these studies often rely on univariate analysis or correlation-based approaches, which fail to identify predictors with robust associations. Additionally, many of the studies included in these reviews based their analyses on data measured at the time of SRNS diagnosis, which may not have accurately captured the predictive potential of biomarkers over time. This approach limited the ability to establish reliable prediction models, as it overlooked the dynamic nature of disease progression, and may have led to overestimation of associations that are not causally linked.

This study aims to comprehensively review existing multivariable prediction models for SRNS and assess their methodological quality. It also summarizes reported risk factors associated with SRNS to inform candidate predictor selection in future prediction model development.

## 2. Materials and Methods

This review was prospectively registered with PROSPERO (CRD420250651875). Reporting adhered to the Transparent Reporting of a Multivariable Prediction Model for Individual Prognosis or Diagnosis for Systematic Reviews and Meta-Analysis [10], as well as the Preferred Reporting Items for Systematic Reviews and Meta-Analyses [11]. The details of self-reports are provided in Supplementary Tables S1 and S2.

### 2.1. Searching Strategies

Two researchers (YH. Hu and ZH. Zhang) independently searched the China National Knowledge Infrastructure, the Chinese Biomedical Literature Database, Wanfang Database, VIP Database, PubMed, Embase, and Scopus databases. We searched for studies developing prediction models for SRNS or identifying associated risk factors. Search terms included “Nephrotic Syndrome”, “Resistan*”, “Steroid”, “Predict*”, and “Risk*”. The full search strategies are presented in Supplementary Table S3. Searches were conducted without language restrictions and included records from the inception of each database through 1 March 2025.

### 2.2. Inclusion and Exclusion Criteria

For predictive models, we included studies that described the development or validation of multivariable SRNS prediction models in children. Eligibility was determined according to the Transparent Reporting of a multivariable prediction model for Individual Prognosis or Diagnosis statement [12] for transparent reporting of multivariable prediction model studies.

Inclusion criteria include: (1) Study population: Children diagnosed with primary nephrotic syndrome, regardless of initial diagnosis or recurrence status, and with no restrictions on age, gender, or race; SRNS was defined as persistent proteinuria after 4–8 weeks of adequate full-dose prednisone or prednisolone therapy [1,4]; (2) Intervention: Each included study reported the development or external validation of a prediction model for glucocorticoid response in pediatric patients with nephrotic syndrome; (3) Comparison: Not performed, as no predictive model has been widely validated across multiple studies; (4) Outcome: Glucocorticoid treatment response; (5) Study design: We included cohort studies (prospective or retrospective), case-control studies, randomized controlled trials, and cross-sectional studies in this review; (6) Time: All predictors must be measured prior to the assessment of glucocorticoid treatment response, specifically at the initial diagnosis of nephrotic syndrome. Exclusion criteria include: (1) Studies solely focusing on genetic or renal biopsy indicators, due to the KDIGO guidelines explicitly stating that routine genetic testing or renal biopsy is not recommended for newly diagnosed patients [4]; (2) Studies collecting or measuring variables after confirmation of glucocorticoid treatment response; (3) Preprints; (4) duplicate publications and studies were not available.

For risk factors, we included studies that examined predictors of glucocorticoid response in children with primary nephrotic syndrome using multivariable regression analysis following univariate screening. Like the predictive models, risk factors must also be assessed before the measurement of glucocorticoid treatment response. No limitations were applied regarding study design, and the exclusion criteria were the same as those used for prediction model studies.

### 2.3. Selection of Reviews

Two investigators (YH. Hu and ZH. Zhang) independently screened titles and abstracts, and subsequently reviewed the full texts of potentially eligible studies to assess which articles would be included. Discrepancies were resolved by a third researcher (HL. Li) through a consensus discussion.

### 2.4. Data Extraction

Two authors (YH. Hu and ZH. Zhang) independently performed data extraction, and any discrepancies were resolved by a third author (HL. Li). The following data were extracted from all included studies: first author, publication year, country/region, and model name (where applicable). Corresponding authors were contacted to obtain missing information/data. For predictive model research, a standardized form was constructed using the CHARMS checklist [13]. The extracted information from studies reporting model development included study design, study population, outcome measures, candidate predictors, modeling approach, internal validation method, numbers of patients and events, handling of missing and continuous variables, number and type of predictors in the final model, model presentation, and performance metrics (classification, discrimination, calibration, and clinical utility). Classification performance was assessed by sensitivity, specificity, positive predictive value, and negative predictive value; discrimination was measured using the C-index; calibration was examined with the Hosmer-Lemeshow test and calibration plots; and clinical utility was evaluated through decision curve analysis and net benefit metrics. For external validation studies, extracted information included study population, number of patients and events, and measures of predictive performance. For studies reporting multiple models, information was extracted independently for each individual model. When studies reported both overall and subgroup-specific model performance, only overall population analyses were extracted.

For risk factor studies, the extracted information included the study design, study population, number of patients, analysis methods, and the results of the analysis.

### 2.5. Assessment of Methodological Quality of Included Studies

Two authors (YH. Hu and ZH. Zhang) independently evaluated the risk of bias for the included prediction models using the Prediction Model Risk of Bias Assessment Tool (PROBAST) [14], and similarly applied the Newcastle-Ottawa Scale [15] to the observational studies. Any discrepancies between reviewers were resolved through discussion with a third author (HL. Li).

### 2.6. Data Synthesis and Analysis

Baseline characteristics were summarized using mean ± standard deviation or median (interquartile range, IQR) for continuous variables, and expressed as percentages for categorical variables. For prediction models that had been validated in more than two independent datasets, we conducted a random-effects meta-analysis using the area under the curve (AUC) and 95% confidence intervals (CI) as the primary metrics. A 95% CI including 0.5 was considered indicative of insufficient discrimination. For risk factors, we applied a random-effects meta-analysis to combine odds ratios (OR) with their corresponding 95% CI. Heterogeneity (*I*^2^) was assessed only for pooled analyses. Funnel plots were generated to evaluate publication bias when ten or more studies reported comparable outcomes. Two-tailed statistical tests were conducted with a significance level of *p* < 0.05. All analyses were performed using SPSS version 18.0 and RevMan version 5.3.

### 2.7. Sensitivity Analysis

Sensitivity analyses were planned separately for prediction model studies and risk factor studies. For prediction model studies, if the same model was externally validated in more than five independent datasets, sensitivity analysis would be conducted by excluding studies at high risk of bias and re-pooling the model performance estimates. For risk factor studies, if at least five studies reported the same risk factor with comparable definitions, sensitivity analysis would be conducted by excluding studies at high risk of bias and re-pooling the effect estimates.

## 3. Results

A total of 2264 records were retrieved. After screening titles and abstracts, 23 unique studies were included, comprising 13 predictive model studies and 19 risk factor studies with an overlap of nine studies between the two categories. This overlap is attributed to the fact that these nine studies developed predictive models by adopting multivariate analysis methods to screen and incorporate key risk factors.

**Figure 1:**
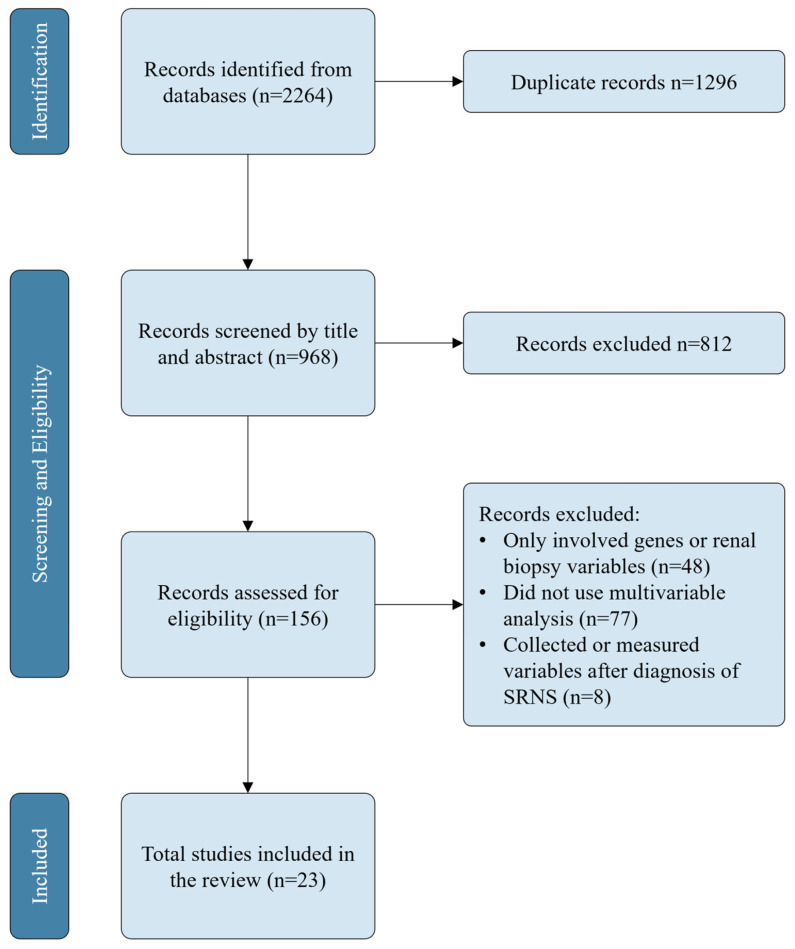
Flow diagram of study selection.

The literature screening process is presented in Figure 1.

### 3.1. Results of Prediction Models

Thirteen studies encompassed the development of 17 prediction models and the external validation of one model. Model development occurred primarily in China (*n* = 9), followed by the United States (*n* = 6). All development and validation utilized retrospective electronic health records from hospitalized cohorts. Across development cohorts, median sample size was 56 (IQR 41.5–116.5), with 30 events per variable (IQR 15.5–38.5) and 8.5 predictors per model (IQR 4.5–15.3). The external validation cohort contained 50 patients with 20 events and the events per variable was 2.5. Eight studies evaluated patients’ glucocorticoid response based on whether urinary protein turned negative after 4 weeks of glucocorticoid administration (Table 1). Forty four predictors were documented. Most frequent predictors included: serum erythrocyte sedimentation rate (ESR) (*n* = 3), vitamin D-binding protein (VDBP) (*n* = 3), age (*n* = 2), serum D-dimer (*n* = 2), serum β2-microglobulin (*n* = 2), serum white blood cell (*n* = 2), serum cholesterol (*n* = 2), serum creatinine (*n* = 2), serum suppressor T cells (*n* = 2), urinary α-1-acid glycoprotein (*n* = 2), urinary retinol-binding protein (*n* = 2), urinary albumin (*n* = 2), urinary fetuin-A (*n* = 2), and urinary neutrophil gelatinase-associated lipocalin (*n* = 2). Supplementary Table S4 summarizes the predictors and performance of each model.

Models were predominantly developed using logistic regression (*n* = 16, 94.1%), followed by machine learning approaches (*n* = 1, 5.9%). Two models were respectively internally validated using bootstrap sampling and cross-validation methods. No model addressed missing values via interpolation. Most models (*n* = 7, 41.2%) directly excluded samples with missing data. All studies reported AUC values ranging from 0.75 to 0.88. Given that no predictive model has been externally validated in more than two cohorts, meta-analysis of AUC was not feasible.

Four models (23.5%) reported calibration via Hosmer-Lemeshow tests, while decision curve analysis evaluated clinical utility in two models (11.8%). The most common model presentation formats were mathematical equations (*n* = 6, 35.3%), followed by nomograms (*n* = 2, 11.8%) and web-based calculators (*n* = 2, 11.8%). One model that underwent external validation within the same healthcare system demonstrated a sensitivity of 0.9, specificity of 0.97, and accuracy of 0.94.

Overall, 76.92% of the models exhibited a high risk of bias (Supplementary Table S5), with the primary source of high risk of bias being the analytical domain (76.92%), particularly due to inadequate sample sizes (46.15%) or improper handling of missing data (38.46%). Details of the PROBAST risk-of-bias assessment are provided in Supplementary Table S6. These findings indicate that the reported model performance estimates are likely to be unstable and potentially optimistic.

### 3.2. Results of Risk Factors

The 19 included studies were published between 2012 and 2024, with the majority conducted in China (*n* = 8). The total number of patients across all studies was 2514. The study designs primarily included case-control studies (*n* = 15), retrospective cohort studies (*n* = 1), and prospective cohort studies (*n* = 3). All studies employed multivariate logistic regression to identify independent risk factors for SRNS. Ten studies evaluated patients’ glucocorticoid response based on whether urinary protein turned negative after 4 weeks of glucocorticoid administration (Table 2).

**Figure 2:**
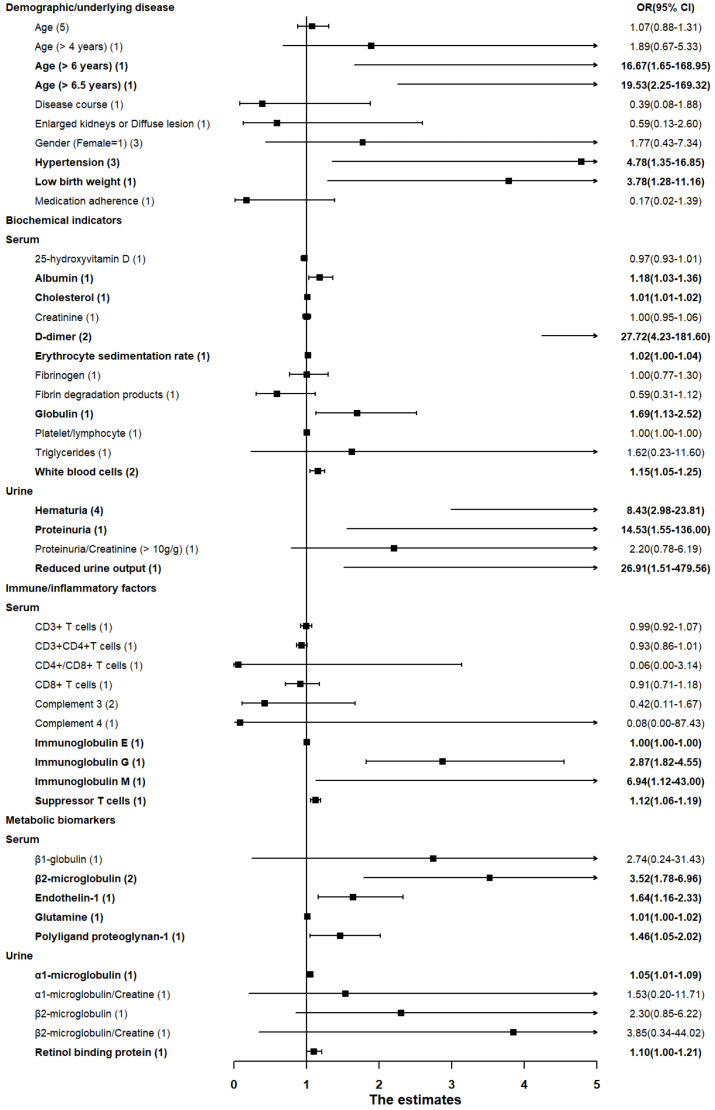
Associations between reported risk factors and SRNS (summary of multivariable results). **Footnote:** Risk factors of demographic/underlying disease, biochemical indicators, immune/inflammatory factors and metabolic biomarkers. Prognostic factor (number of studies) (right column): OR and 95% CI from pooled analysis; Boldface indicates statistically significant associations (*p* < 0.05); For continuous variables, the effect estimates correspond to the unit of increase as reported in the original studies; for categorical variables, the cutoff/definition is indicated in the label.

A total of 52 risk factors were analyzed across all studies, with 22 independent risk factors ultimately identified (Figure 2). The corresponding forest plots are presented in Supplementary Figure S1, and the definition of each pooled risk factor is provided after the relevant forest plot. Only a small subset of these independent risk factors could be quantitatively pooled; the remainder were reported descriptively. The 22 independent risk factors included: demographic/underlying disease-related factors (*n* = 3): age, low birth weight, hypertension; biochemical indicators (*n* = 9): white blood cells, cholesterol, ESR, albumin, D-dimer, globulin, proteinuria, decreased urine output, hematuria; immune/inflammatory factors (*n* = 4): suppressor T cells, immunoglobulin E, immunoglobulin M, immunoglobulin G; metabolic biomarkers (*n* = 6): polyligand proteoglynan-1, glutamine, endothelin-1, retinol-binding protein, α1-microglobulin, β2-microglobulin.

Overall, among the 19 observational studies, 14 were assessed as high-quality and 5 as medium-quality (Supplementary Table S7). None of the pooled analyses included 10 or more studies; therefore, funnel plots were not generated and publication bias could not be formally assessed.

### 3.3. Results of Sensitivity Analysis

Sensitivity analyses were not conducted because the prespecified criteria were not met: prediction models had insufficient external validation studies, and age, the only risk factor reported in at least five studies, included no high-risk-of-bias study for exclusion.

### 3.4. Cross-Comparison and Analysis

**Figure 3:**
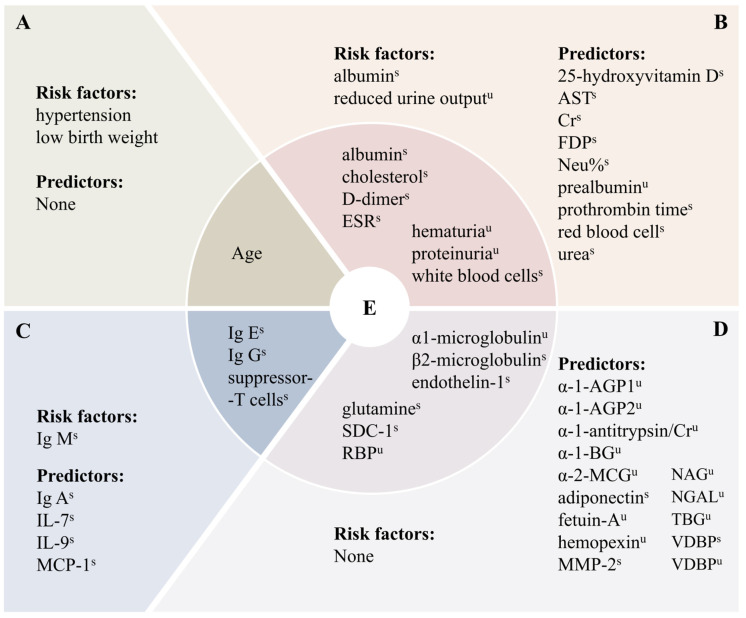
Overview of included risk factors and predictors. **Footnote:** (**A**–**D**): Variables included exclusively of risk factors and predictors in Demographic/underlying disease, Biochemical indicators, Immune/inflammatory factors and Metabolic biomarkers; (**E**): Variables reported as both risk factors and predictors (overlapping variables). **^S^:** measured in serum; **^U^:** measured in urine. **α-1-AGP1:** α-1 acid glycoprotein 1; **α-1-AGP2:** α-1 acid glycoprotein 1; **α-1-BG:** α-1-B glycoprotein; **α-2-MCG:** α-2 macroglobulin; **AST:** aspartate aminotransferase; **Cr:** Creatinine; **ESR:** erythrocyte sedimentation rate; **FDP:** fibrin degradation products; **IgA:** Immunoglobulin A; **IgE:** Immunoglobulin E; **IgG:** Immunoglobulin G; **IgM:** Immunoglobulin M; **IL-7:** interleukin-7; **IL-9:** interleukin-9; **MMP-2:** matrix metalloproteinase 2; **MCP-1:** monocyte chemoattractant protein 1; **NAG:** N-acetyl-β-D-glucosaminidase; **Neu%:** percentage of neutrophils; **NGAL:** neutrophil gelatinase-associated lipocalin; **RBP:** retinol-binding protein; **SDC-1:** polyligand proteoglynan-1; **TBG:** thyroxine-binding globulin; **VDBP:** vitamin D binding protein.

Cross-referencing revealed 17 variables serving as both predictive factors and independent risk factors. In addition, five variables were reported as independent risk factors but have not been incorporated into the published multivariable prediction models, including low birth weight, reduced urine output, hypertension, serum albumin, and serum immunoglobulin M (Figure 3).

## 4. Discussion

This systematic review evaluated 17 predictive models for childhood SRNS and identified 22 associated independent risk factors. Despite the reported apparent discriminative performance (AUC range: 0.75–0.88; AUC > 0.70 indicating acceptable discrimination [38]), these estimates should be interpreted with substantial caution because most models were at high risk of bias, were developed in small retrospective cohorts, and lacked adequate internal or external validation. First, only two models underwent internal validation and one model underwent external validation, which may lead to overestimation of model performance due to overfitting [39] and limit the generalizability of models across populations with different characteristics [40]. Therefore, the reported AUC values should not be interpreted as evidence of reliable clinical prediction; the reliability, robustness, and clinical applicability of existing models remain very limited at present. Second, missing data were often inadequately handled (frequently by complete-case analysis), which may bias estimates and reduce predictive accuracy [41]. Third, calibration was reported in fewer than one-quarter of models, and decision-curve analysis was rarely performed, constraining assessment of clinical usefulness [42]. Fourth, decision curve analysis quantifies clinical net benefit post-implementation [43], yet only two studies implemented this analysis. Finally, many models lacked a clear presentation format, which may impede translation into routine practice [44].

This study found that ESR and VDBP measured at disease onset were the most frequently included predictors in SRNS prediction models. In nephrotic syndrome, ESR is often markedly elevated at presentation, largely due to increased circulating acute-phase reactants (e.g., fibrinogen and other pro-aggregatory proteins) and altered plasma protein composition that promotes erythrocyte aggregation [45,46]. Such elevated baseline ESR may reflect a greater inflammatory burden and more active immune dysregulation at disease onset, underlying its potential predictive value. VDBP may reflect urinary protein loss, tubular injury, or inflammation-related renal damage, which may explain why it has been evaluated as a potential predictor [47,48].

Among variables frequently incorporated during model development, approximately 50% are not readily obtainable through routine clinical laboratory testing. This requirement for specialized reagent procurement consequently constrains their applicability in resource-limited settings, including primary care facilities.

Risk factors should not be considered equivalent to predictors in clinical prediction models, but they may provide clues for identifying candidate predictors that require further validation. This review found that five factors—low birth weight, reduced urine output, hypertension, serum albumin, and serum immunoglobulin M—were reported as independent risk factors but have not yet been incorporated into published multivariable prediction models. Due to the observational design and risk of bias of included studies, these associations are hypothesis-generating. Low birth weight is a well-established risk factor for SRNS, as studies have shown that the incidence of SRNS in low-birth-weight children is 3.78 times that in children with normal birth weight; for every 100 g increase in birth weight, the risk of SRNS decreases by approximately 8% [32]. A potential explanation is that low-birth-weight children, due to incomplete renal development and reduced nephron number, are prone to compensatory impairment of renal function, thereby increasing the risk of SRNS and exhibiting a higher recurrence rate and more complications [49,50,51]. Hypertension is another clinical marker that commonly accompanies nephrotic syndrome. It results from fluid retention due to glomerular damage, which, in turn, exacerbates kidney injury and promotes a vicious cycle of further renal deterioration [52,53]. Reduced urine output may serve as an early warning sign, indicating a decline in renal filtration capacity and worsening renal function, both of which may increase the risk of SRNS progression [31]. These markers not only reflect the severity of NS at diagnosis but also provide critical insight into the potential for steroid resistance, making them useful for early stratification. Evidence from the included multivariable analyses suggested an association between higher serum albumin levels and SRNS. This finding should be interpreted cautiously because the direction and mechanism of this association remain uncertain. Serum albumin may reflect differences in disease phenotype, proteinuria selectivity [7,54], or underlying histopathology [55], but its value as a candidate predictor requires further validation. In this review, higher serum IgM was identified as an independent risk factor for SRNS. Although biopsy-derived predictors were outside our eligibility criteria, biopsy-based studies have reported that glomerular immunoglobulin M correlates with the severity of kidney injury, suggesting an elevated risk of disease recurrence and protracted remission periods. Furthermore, children with immunoglobulin M positivity demonstrate enhanced therapeutic responsiveness to cyclosporine treatment, which provides further evidence for the role of immune factors in steroid resistance [56,57]. Finally, descriptive analyses in several studies identified additional potential independent risk factors, including pathological types, peripheral blood CD4^+^T cell levels, and serum macrophage migration inhibitory factor, which were not represented among predictors in the published multivariable models included in this review.

The primary strength of this study is its systematic synthesis and evaluation of childhood SRNS prediction models, as well as the identification of independent risk factors associated with SRNS development, thereby providing an evidence-based reference for optimizing future prediction models. However, this study has several limitations. First, most prediction model studies were at high risk of bias, mainly due to limitations in the analysis domain, including small sample sizes, limited validation, and inadequate handling of missing data. Therefore, the reported model performance and identified predictors should be interpreted cautiously. Second, due to the scarcity of externally validated prediction models, meta-analysis and sensitivity analysis of model performance could not be performed. Third, relatively few studies were available for each risk factor, and most associations were supported by only one or two studies, limiting the robustness of pooled estimates and precluding sensitivity analyses. Finally, substantial heterogeneity in SRNS definitions, populations, laboratory methods, and adjustment models may have affected the reliability of the synthesized findings.

## 5. Conclusions

This systematic review summarized multivariable prediction models and independent risk factors for childhood SRNS. Although existing models reported apparent discrimination, most had a high risk of bias, lacked external validation, and included predictors that are not routinely available in clinical practice, indicating that their current clinical applicability remains very limited. The identified risk factors may provide preliminary clues for future candidate predictor selection, but their incremental predictive value and clinical feasibility require further evaluation. Future studies should prioritize clinically accessible predictors, rigorous model development, multi-center external validation, calibration assessment, decision curve analysis, and interpretable model presentation to improve clinical utility and generalizability.

## Figures and Tables

**Table 1:** Basic characteristics of the included studies on SRNS prediction models.

Study	Country	Study Aim	Cohort Type	SR Cases/Total Patients	Age (Years)	Outcome Definition
Agrawal 2021 [16]	USA	D	EHR	14/40	7.0 (mean)	NP-7 weeks
Agrawal 2020 [17]	USA	D	EHR	13/37	8.7 (mean)	NP-7 weeks
Bennett 2017 [18]	USA	D	EHR	20/50	12.3 (mean)	NP-8 weeks
Burlaka 2022 [19]	Ukraine	D	EHR	30/56	11.0 (mean)	NP-6 weeks
Gooding 2020 [20]	USA	D	EHR	14/39	9.5 (mean)	NP-6 weeks
Hong 2022 [21]	China	D	EHR	41/174	3.0 (median)	NP-4 weeks
Jiang 2019 [22]	China	D	EHR	16/79	5.5 (mean)	NP-4 weeks
Kou 2023 [23]	China	D	EHR	65/111	3.2 (median)	NP-4 weeks
Ling 2019 [24]	China	D	EHR	44/122	5.8 (mean)	NP-4 weeks
Liu 2023 [25]	China	D	EHR	31/157	9.0 (mean)	NP-4 weeks
Wang 2012 [26]	China	D	EHR	19/43	4.5 (mean)	NP-4 weeks
Ye 2023 [5]	China	D, EV	EHR	34/91, 20/50	4.0 (median)	NP-4 weeks
Zhang 2015 [27]	China	D	EHR	36/185	6.0 (median)	NP-4 weeks

**Footnote: D:** derivation; **EHR:** electronic health record; **EV:** external validation; **NP:** Negative Proteinuria.

**Table 2:** Basic characteristics of the included studies on SRNS-associated risk factors.

Study	Country	Study Design	Number of Participants	Outcome Definitions	Independent Risk Factors
Agrawal 2021 [16]	USA	R	40	NP-7 weeks	Serum: interleukin-7, interleukin-9, monocyte chemoattractant protein 1
Agrawal 2020 [17]	USA	R	37	NP-7 weeks	Serum: adiponectin, matrix metalloproteinase 2, vitamin D binding protein
Cicek 2024 [28]	Turkey	R	162	NP-8 weeks	Age Urine: microscopic hematuria
Cuzzoni 2019 [29]	Italy	P	62	NP-4 weeks	Age Serum: macrophage migration inhibitory factor
Gooding 2020 [20]	USA	R	39	NP-6 weeks	Age Serum: glutamine
Hong 2022 [21]	China	C	174	NP-4 weeks	Serum: white blood cell Urine: retinol-binding protein
Imbusi 2020 [30]	Ethiopia	C	63	NP-8 weeks	Age (>6), hypertension Serum: cholesterol
Kifle 2020 [31]	Ethiopia	C	85	NP-4 weeks	Urine: gross hematuria, urine output
Konstantelos 2019 [32]	Canada	P	377	Not reported	low birth weight
Kou 2023 [23]	China	C	111	NP-4 weeks	Serum: β-microglobulin, D-dimer, erythrocyte sedimentation rate, suppressor T cells
Li 2024 [33]	China	C	510	NP-8 weeks	Pathology, age Serum: 25-hydroxyvitamin D, CD4+ T cells, creatinine, D-dimer Urine: proteinuria
Ling 2019 [24]	China	C	122	NP-4 weeks	Serum: immunoglobulin E, immunoglobulin G
Liu 2023 [25]	China	C	157	NP-4 weeks	Serum: endothelin-1, polyligandproteoglynan-1
Rehman 2022 [34]	Pakistan	R	135	Not reported	Hypertension Urine: hematuria
Salah 2023 [35]	Egypt	P	60	NP-8 weeks	Age Serum: white blood cell
Udagawa 2021 [36]	Japan	C	80	NP-4 weeks	Gender Serum: immunoglobulin M
Wang 2012 [26]	China	C	43	NP-4 weeks	Serum: globulin Urine: α1-antitrypsin/creatinine, αl-microglobulin
Yin 2010 [37]	China	C	72	NP-4 weeks	Age Serum: albumin, D-dimer
Zhang 2015 [27]	China	C	185	NP-4 weeks	Age (>6.5) Urine: microscopic hematuria, proteinuria,

**Footnote: C:** Case-control study; **NP:** Negative Proteinuria; **P:** Prospective cohort study; **R:** Retrospective cohort study.

## Data Availability

The datasets used and analyzed during the current study are available in the Supplementary Materials.

## Supplementary Materials

The following supporting information can be downloaded at: https://www.mdpi.com/article/10.3390/jcm15124438/s1. (1) Supplementary Table S1: TRIPOD-SRMA Checklist for reporting systematic reviews of prediction model studies; (2) Supplementary Table S2: PRISMA Checklist for reporting systematic reviews and Meta-analysis; (3) Supplementary Table S3: Search Strategy; (4) Supplementary Table S4: Outcomes of studies reporting on prediction models; (5) Supplementary Table S5: Risk of bias and applicability assessment for each PROBAST domain; (6) Supplementary Table S6: Details of PROBAST risk-of-bias assessment; (7) Supplementary Table S7: Risk of bias and applicability assessment for each NOS domain; (8) Supplementary Figure S1: Forest plots for pooled analyses of risk factors associated with steroid-resistant nephrotic syndrome in children.

## Abbreviations

**AUC:** area under the curve; **CHARMS:** Critical Appraisal and Data Extraction for Systematic Reviews of Prediction Modelling Studies; **CI:** Confidence Interval; **ESR:** erythrocyte sedimentation rate; **IQR:** Interquartile Range; **KDIGO:** Kidney Disease: Improving Global Outcomes; **OR:** odds ratios; **PNS:** Primary Nephrotic Syndrome; **PROBAST:** prediction model risk of bias assessment tool; **SRNS:** Steroid-Resistant Nephrotic Syndrome; **TRIPOD:** Transparent Reporting of a multivariable prediction model for Individual Prognosis or Diagnosis; **VDBP:** vitamin D-binding protein.

## References

[1] Noone D.G., Iijima K., Parekh R.J.T.L. (2018). Idiopathic nephrotic syndrome in children. Lancet.

[2] Mendonça A.C., Oliveira E.A., Fróes B.P., Faria L.D., Pinto J.S., Nogueira M.M., Lima G.O., Resende P.I., Assis N.S., Simões E.S.A.C. (2015). A predictive model of progressive chronic kidney disease in idiopathic nephrotic syndrome. Pediatr. Nephrol..

[3] Trautmann A., Schnaidt S., Lipska-Ziętkiewicz B.S., Bodria M., Ozaltin F., Emma F., Anarat A., Melk A., Azocar M., Oh J. (2017). Long-Term Outcome of Steroid-Resistant Nephrotic Syndrome in Children. J. Am. Soc. Nephrol..

[4] Trautmann A., Vivarelli M., Samuel S., Gipson D., Sinha A., Schaefer F., Hui N.K., Boyer O., Saleem M.A., Feltran L. (2020). IPNA clinical practice recommendations for the diagnosis and management of children with steroid-resistant nephrotic syndrome. Pediatr. Nephrol..

[5] Ye Q., Li Y., Liu H., Mao J., Jiang H. (2023). Machine learning models for predicting steroid-resistant of nephrotic syndrome. Front. Immunol..

[6] Zaorska K., Zawierucha P., Świerczewska M., Ostalska-Nowicka D., Zachwieja J., Nowicki M. (2021). Prediction of steroid resistance and steroid dependence in nephrotic syndrome children. J. Transl. Med..

[7] Floege J., Gibson K.L., Vivarelli M., Liew A., Radhakrishnan J., Rovin B.H. (2025). KDIGO 2025 Clinical Practice Guideline for the Management of Nephrotic Syndrome in Children. Kidney Int..

[8] May C.J., Ford N.P., Welsh G.I., Saleem M.A. (2025). Biomarkers to predict or measure steroid resistance in idiopathic nephrotic syndrome: A systematic review. PLoS ONE.

[9] Lee J.M., Ahn Y.H., Lim S.H., Kang H.G. (2021). Biomarkers predicting treatment-response in nephrotic syndrome of children: A systematic review. Child. Kidney Dis..

[10] Snell K.I.E., Levis B., Damen J.A.A., Dhiman P., Debray T.P.A., Hooft L., Reitsma J.B., Moons K.G.M., Collins G.S., Riley R.D. (2023). Transparent reporting of multivariable prediction models for individual prognosis or diagnosis: Checklist for systematic reviews and meta-analyses (TRIPOD-SRMA). BMJ (Clin. Res. Ed.).

[11] Page M.J., McKenzie J.E., Bossuyt P.M., Boutron I., Hoffmann T.C., Mulrow C.D., Shamseer L., Tetzlaff J.M., Akl E.A., Brennan S.E. (2021). The PRISMA 2020 statement: An updated guideline for reporting systematic reviews. BMJ (Clin. Res. Ed.).

[12] Collins G.S., Reitsma J.B., Altman D.G., Moons K.G. (2015). Transparent reporting of a multivariable prediction model for individual prognosis or diagnosis (TRIPOD): The TRIPOD statement. BMJ (Clin. Res. Ed.).

[13] Moons K.G., de Groot J.A., Bouwmeester W., Vergouwe Y., Mallett S., Altman D.G., Reitsma J.B., Collins G.S. (2014). Critical appraisal and data extraction for systematic reviews of prediction modelling studies: The CHARMS checklist. PLoS Med..

[14] Wolff R.F., Moons K.G.M., Riley R.D., Whiting P.F., Westwood M., Collins G.S., Reitsma J.B., Kleijnen J., Mallett S. (2019). PROBAST: A Tool to Assess the Risk of Bias and Applicability of Prediction Model Studies. Ann. Intern. Med..

[15] Wells G.A., Shea B., O’Connell D., Peterson J., Welch V., Losos M., Tugwell P. The Newcastle-Ottawa Scale (NOS) for Assessing the Quality of Nonrandomised Studies in Meta-Analyses. https://ohri.ca/en/who-we-are/core-facilities-and-platforms/ottawa-methods-centre/newcastle-ottawa-scale.

[16] Agrawal S., Brier M.E., Kerlin B.A., Smoyer W.E. (2021). Plasma Cytokine Profiling to Predict Steroid Resistance in Pediatric Nephrotic Syndrome. Kidney Int. Rep..

[17] Agrawal S., Merchant M.L., Kino J., Li M., Wilkey D.W., Gaweda A.E., Brier M.E., Chanley M.A., Gooding J.R., Sumner S.J. (2020). Predicting and Defining Steroid Resistance in Pediatric Nephrotic Syndrome Using Plasma Proteomics. Kidney Int. Rep..

[18] Bennett M.R., Pleasant L., Haffner C., Ma Q., Haffey W.D., Ying J., Wagner M., Greis K.D., Devarajan P. (2017). A Novel Biomarker Panel to Identify Steroid Resistance in Childhood Idiopathic Nephrotic Syndrome. Biomark. Insights.

[19] Burlaka I., Mityuryayeva I., Bagdasarova I. (2022). Clinical and Apoptotic Factors Defining and Predicting Steroid Resistance in Nephrotic Syndrome in Children. Glob. Pediatr. Health.

[20] Gooding J.R., Agrawal S., McRitchie S., Acuff Z., Merchant M.L., Klein J.B., Smoyer W.E., Sumner S.J. (2020). Predicting and Defining Steroid Resistance in Pediatric Nephrotic Syndrome Using Plasma Metabolomics. Kidney Int. Rep..

[21] Hong M.Z., Zhong P.Q., Chen M.Y., Chen P.S. (2022). Laboratory indicators predict the efficacy of glucocorticoid therapy in pediatric primary nephrotic syndrome. J. Trop. Med..

[22] Jiang Y., Zhang B.L., Wang W.H. (2019). Clinical significance of detection of urine renal injury biomarkers in children with primary nephrotic syndrome. Chin. J. Appl. Clin. Pediatr..

[23] Kou M., Wu F., Qu X.Y., Wang H., Guo X.T., Yang Y.Y., Zhao L.J. (2023). Establishment and validation of clinical prediction model for steroid-resistant nephrotic syndrome in children. Chin. J. Pediatr..

[24] Ling C., Chen Z., Fan J.F., Sun Q., Meng Q., Hua L., Liu X.R. (2019). Value of serum lgG combined with IgE in predicting steroid therapy response in children with primary nephrotic syndrome. Chin. J. Nephrol..

[25] Liu Q., Zhu J., Tang G.Y., Jiang P.Y. (2023). Serum levels of ET-1 and SDC-1 in children with nephrotic syndrome and their correlation with hormone therapy response. Tianjin Med. J..

[26] Wang Y.Y., DIng G.X., Yuan Y.G., Bao H.Y., Chen Y., Zhao F., Han Y., Zhang A.H., Huang S.M. (2012). Detection of urinary α1-antitrypsin for predicting response to glucocorticoid therapy in children with primary nephrotic syndrome. Chin. J. Nephrol..

[27] Zhang B.L., Liu T., Lin S.X., Wang W.H., Liu Y., Liu Y., Wu X., Wang X., Liu Z. (2015). Analysis of risk factors for steroid resistance in children with primary nephritic syndrome and discussion on the prediction model of steroid resistance. Chin. J. Nephrol..

[28] Cicek N., Yıldız N., Guven S., Kaya M., Gokce I., Alpay H. (2024). Clinical Predictors of Steroid Resistance in Childhood Nephrotic Syndrome. Clin. Pediatr..

[29] Cuzzoni E., Franca R., De Iudicibus S., Marcuzzi A., Lucafò M., Pelin M., Favretto D., Monti E., Morello W., Ghio L. (2019). MIF plasma level as a possible tool to predict steroid responsiveness in children with idiopathic nephrotic syndrome. Eur. J. Clin. Pharmacol..

[30] Imbusi E.A., Ekanem P.E., Gebrearegay H., Ambaye M., Tesfahunegn A., Nyaga K., Ekanem R., Peter N. (2020). Steroid response pattern among children with nephrotic syndrome in Northern Ethiopia. Nephro-Urol. Mon..

[31] Kifle M., Shimelis D. (2020). Predictors of resistance to steroids in pediatric nephrotic syndrome at a tertiary hospital, Addis Ababa. Ethiop. J. Pediatr. Child Health.

[32] Konstantelos N., Banh T., Patel V., Vasilevska-Ristovska J., Borges K., Hussain-Shamsy N., Noone D., Hebert D., Radhakrishnan S., Licht C.P.B. (2019). Association of low birth weight and prematurity with clinical outcomes of childhood nephrotic syndrome: A prospective cohort study. Pediatr. Nephrol..

[33] Li J.Z., Wang Y. (2024). Influencing factors and hormone resistance of 510 children with nephrotic syndrome. J. Public Health Prev. Med..

[34] Rehman M., Ali A., Ehsan A., Aziz M., Khatri S., Hashmi S. (2022). Can Steroid Response in Idiopathic Childhood Nephrotic Syndrome be Predicted? A Single Center Quasi-Experimental Study. Pak. Armed Forces Med. J..

[35] Salah D.M., Aoun A.H., Fahmy B.S., Zeid A., Fahmy Y.A. (2023). Does Urinary Vitamin D-Binding Protein Have a Role in the Prediction of Steroid Resistance in Nephrotic Syndrome? A Cohort Study on Egyptian Children. J. Compr. Pediatr..

[36] Udagawa T., Matsuyama Y., Okutsu M., Motoyoshi Y., Okada M., Tada N., Kikuchi E., Shimoda M., Kanamori T., Omori T. (2021). Association between Immunoglobulin M and Steroid Resistance in Children with Nephrotic Syndrome: A Retrospective Multicenter Study in Japan. Kidney360.

[37] Yin L., Zhou W., Sun H., Jin Y.L., Zhou Z.Y. (2010). Risk Factors of Steroid Resistance in Children with Primary Nephrotic Syndrome. Chin. J. Appl. Clin. Pediatr..

[38] White N., Parsons R., Collins G., Barnett A. (2023). Evidence of questionable research practices in clinical prediction models. BMC Med..

[39] Moons K.G., Kengne A.P., Woodward M., Royston P., Vergouwe Y., Altman D.G., Grobbee D.E. (2012). Risk prediction models: I. Development, internal validation, and assessing the incremental value of a new (bio)marker. Heart (Br. Card. Soc.).

[40] Moons K.G., Kengne A.P., Grobbee D.E., Royston P., Vergouwe Y., Altman D.G., Woodward M. (2012). Risk prediction models: II. External validation, model updating, and impact assessment. Heart (Br. Card. Soc.).

[41] Little R.J. (2024). Missing Data Analysis. Annu. Rev. Clin. Psychol..

[42] Alba A.C., Agoritsas T., Walsh M., Hanna S., Iorio A., Devereaux P.J., McGinn T., Guyatt G. (2017). Discrimination and Calibration of Clinical Prediction Models: Users’ Guides to the Medical Literature. JAMA.

[43] Vickers A.J., Van Calster B., Steyerberg E.W. (2016). Net benefit approaches to the evaluation of prediction models, molecular markers, and diagnostic tests. BMJ (Clin. Res. Ed.).

[44] Bonnett L.J., Snell K.I.E., Collins G.S., Riley R.D. (2019). Guide to presenting clinical prediction models for use in clinical settings. BMJ (Clin. Res. Ed.).

[45] Fabry T.L. (1987). Mechanism of erythrocyte aggregation and sedimentation. Blood.

[46] Higuchi M., Watanabe N. (2023). Determination of the erythrocyte sedimentation rate using the hematocrit-corrected aggregation index and mean corpuscular volume. J. Clin. Lab. Anal..

[47] Bouillon R., Schuit F., Antonio L., Rastinejad F. (2019). Vitamin D Binding Protein: A Historic Overview. Front. Endocrinol..

[48] Mirković K., Doorenbos C.R., Dam W.A., Lambers Heerspink H.J., Slagman M.C., Nauta F.L., Kramer A.B., Gansevoort R.T., van den Born J., Navis G. (2013). Urinary vitamin D binding protein: A potential novel marker of renal interstitial inflammation and fibrosis. PLoS ONE.

[49] Teeninga N., Schreuder M.F., Bökenkamp A., Delemarre-van de Waal H.A., van Wijk J.A. (2008). Influence of low birth weight on minimal change nephrotic syndrome in children, including a meta-analysis. Nephrol. Dial. Transplant. Off. Publ. Eur. Dial. Transpl. Assoc.—Eur. Ren. Assoc..

[50] Plank C., Ostreicher I., Dittrich K., Waldherr R., Voigt M., Amann K., Rascher W., Dötsch J. (2007). Low birth weight, but not postnatal weight gain, aggravates the course of nephrotic syndrome. Pediatr. Nephrol..

[51] Conti G., De Vivo D., Fede C., Arasi S., Alibrandi A., Chimenz R., Santoro D. (2018). Low birth weight is a conditioning factor for podocyte alteration and steroid dependance in children with nephrotic syndrome. J. Nephrol..

[52] Shatat I.F., Becton L.J., Woroniecki R.P. (2019). Hypertension in Childhood Nephrotic Syndrome. Front. Pediatr..

[53] Skrzypczyk P., Wabik A.M., Deja A., Ofiara A., Szyszka M., Panczyk-tomaszewska M. (2024). The prevalence and risk factors for arterial hypertension in children with idiopathic nephrotic syndrome. J. Hypertens..

[54] Tojo A. (2019). Mechanism Underlying Selective Albuminuria in Minimal Change Nephrotic Syndrome. Int. J. Nephrol..

[55] Haeri H.H., Eisermann J., Schimm H., Büscher A., Hoyer P., Hinderberger D. (2023). Profound Changes in Functional Structure and Dynamics of Serum Albumin in Children with Nephrotic Syndrome: An Exploratory Research Study. J. Med. Chem..

[56] Swartz S.J., Eldin K.W., Hicks M.J., Feig D.I. (2009). Minimal change disease with IgM+ immunofluorescence: A subtype of nephrotic syndrome. Pediatr. Nephrol..

[57] Kanemoto K., Ito H., Anzai M., Matsumura C., Kurayama H. (2013). Clinical significance of IgM and C1q deposition in the mesangium in pediatric idiopathic nephrotic syndrome. J. Nephrol..

