# Supplementary material for: Prediction Models and Risk Factors for Steroid Resistance in Children with Nephrotic Syndrome: A Systematic Review and Meta-Analysis
Source: J Clin Med. 2026 Jun 8;15(12):4438. doi: 10.3390/jcm15124438 (PMC13301261; doi:10.3390/jcm15124438)
Supplement: Supplementary file 1 [file jcm-15-04438-s001.zip › jcm-4287741-supplementary.pdf]

Supplementary Table 1: TRIPOD-SRMA Checklist for reporting systematic reviews of prediction model studies

Section and topic	Item No	Checklist item	Page
**Title**			
Title	1	Identify the report as a systematic review or meta-analysis (or both) of diagnostic or prognostic model studies. Specify the target population and outcome(s) predicted as relevant to the review question.	1
**Abstract**			
Abstract	2	See the TRIPOD-SRMA Checklist for Abstracts	1
**Introduction**			
Rationale	3	Describe the rationale for the review in the context of existing knowledge.	2-3
Objectives	4	Provide an explicit statement of the objective(s) being addressed with reference to: target population, index and comparator models (as relevant), outcome(s), time (prediction horizon and intended moment of using the model), and setting.	3
**Methods**			
Study eligibility criteria	5	Specify study characteristics used as eligibility criteria, including any prediction models of specific interest, and whether development or validation studies (or both) were eligible.	3-4
Information sources	6	Specify all databases, registers, websites, organisations, reference lists and other sources searched or consulted to identify studies. Specify the date when each source was last searched or consulted.	3
Search strategy	7	Present the full search strategies for all databases, registers and websites, including any filters and limits used.	3
Study selection process	8	Specify the methods used to decide whether a study met the inclusion criteria of the review, including how many reviewers screened each record and each report retrieved, whether they worked independently, and if applicable, details of automation tools used in the process.	4
Data collection process	9	Specify the methods used to collect data from study reports, including how many reviewers collected data from each report, whether they worked independently, any processes for obtaining or confirming data from study investigators, and if applicable, details of automat ion tools used in the process.	4
Data Items	10a	List and define all items for which data were sought from each study.	4
	10b	State the model performance measures that were sought (e.g., measures of calibration, discrimination, overall model fit, clinical utility).	4
	10c	Describe how any desired but unreported data items (items 10a, 10b) were handled (e.g., contacted authors, calculated from other reported information).	4
Risk of bias and applicability assessment	11	Specify the methods used to assess risk of bias in the included studies and their applicability to the review question. This should be done separately for each model development and validation. Include details of any tool(s) used, how many reviewers assessed each study and whether they worked independently.	4
Synthesis methods	12a	Describe any methods for synthesizing estimates of performance measures for each model. If meta-analysis was carried out, describe the methods used, including any transformations of data prior to pooling, how any heterogeneity in model performance was quantified and handled, and software package(s) used.	4-5
	12b	Describe any methods used to explore possible causes of heterogeneity in model performance (e.g., subgroup analysis, meta-regression), including whether or not they were planned.	4-5

Section and topic	Item No	Checklist item	Page
	12c	Describe any sensitivity analyses conducted to assess robustness of the synthesised results.	4-5
Certainty assessment	13	Describe any methods used to assess certainty (or confidence) in the body of evidence for a prediction model.	NA
**Results**			
Study selection	14	Describe the results of the search and selection process, from the number of records identified in the search to the number of studies and models included in the review, ideally using a flow diagram.	5-6
Study and model characteristics	15	Present study characteristics and model details extracted (as per Item 10a), and cite the study reports.	6-8
Risk of bias and applicability	16	Present results of risk of bias and applicability assessment. This should be done separately for each model development and validation in each included study.	7
Results of model performance in individual studies	17	Present performance estimates and confidence intervals for each model and all evaluations, including whether they relate to the internal or external validation performance. If internal, give details of the method.	7
Results of syntheses	18a	Present the results of any synthesis of model performance, together with details of which study estimates contributed. If meta-analysis was carried out, then for each model and performance measure, present summary results, confidence/credible intervals and measures of heterogeneity. Forest plots may be useful.	NA
	18b	For each model, present results of all investigations of possible causes of heterogeneity in model performance.	NA
	18c	Present results of all sensitivity analyses conducted to assess the robustness of the synthesised results.	7
Certainty of evidence	19	Present any assessments of certainty (or confidence) in the body of evidence for each prediction model of interest.	NA
**Discussion**			
Summary of evidence	20	Summarise the main findings including the strengths and limitations of the evidence.	10-11
Limitations	21	Discuss the strengths and limitations of the review process.	12
Implications	22	Discuss implications of the results in the context of other evidence and for practice, policy, and future research.	12
**Other information**			
Registration and protocol	23a	Provide registration information for the review, including register name and registration number, or state that the review was not registered.	3
	23b	Indicate where the review protocol can be accessed, or state that a protocol was not prepared.	3
	23c	Describe and explain any amendments to information provided at registration or in the protocol.	NA
Support	24	Describe sources of financial or non-financial support for the review, and the role of the funders or sponsors in the review.	13
Competing interests	25	Declare any competing interests of review authors.	13

Section and topic	Item No	Checklist item	Page
Availability of data, code, and other materials	26	Report which of the following are publicly available and where they can be found: template data collection forms; data extracted from included studies; data used for all analyses; analytic code; any other materials used in the review.	13

This checklist appears in appendix 2 of Snell KIE, Levis B, Damen JAA, et al. Transparent reporting of multivariable prediction models for individual prognosis or diagnosis: checklist for systematic reviews and meta-analyses (TRIPOD-SRMA). *BMJ* 2023;381:e073538. doi:10.1136/bmj-2022-073538.

Supplementary Table 2: PRISMA Checklist for reporting systematic reviews and Meta-analysis

Section and Topic	Item #	Checklist item	Location where item is reported
**TITLE**			
Title	1	Identify the report as a systematic review.	1
**ABSTRACT**			
Abstract	2	See the PRISMA 2020 for Abstracts checklist.	1
**INTRODUCTION**			
Rationale	3	Describe the rationale for the review in the context of existing knowledge.	2-3
Objectives	4	Provide an explicit statement of the objective(s) or question(s) the review addresses.	3
**METHODS**			
Eligibility criteria	5	Specify the inclusion and exclusion criteria for the review and how studies were grouped for the syntheses.	3-4
Information sources	6	Specify all databases, registers, websites, organisations, reference lists and other sources searched or consulted to identify studies. Specify the date when each source was last searched or consulted.	3
Search strategy	7	Present the full search strategies for all databases, registers and websites, including any filters and limits used.	3
Selection process	8	Specify the methods used to decide whether a study met the inclusion criteria of the review, including how many reviewers screened each record and each report retrieved, whether they worked independently, and if applicable, details of automation tools used in the process.	4
Data collection process	9	Specify the methods used to collect data from reports, including how many reviewers collected data from each report, whether they worked independently, any processes for obtaining or confirming data from study investigators, and if applicable, details of automation tools used in the process.	5
Data items	10a	List and define all outcomes for which data were sought. Specify whether all results that were compatible with each outcome domain in each study were sought (e.g. for all measures, time points, analyses), and if not, the methods used to decide which results to collect.	4

Section and Topic	Item #	Checklist item	Location where item is reported
	10b	List and define all other variables for which data were sought (e.g. participant and intervention characteristics, funding sources). Describe any assumptions made about any missing or unclear information.	4
Study risk of bias assessment	11	Specify the methods used to assess risk of bias in the included studies, including details of the tool(s) used, how many reviewers assessed each study and whether they worked independently, and if applicable, details of automation tools used in the process.	4
Effect measures	12	Specify for each outcome the effect measure(s) (e.g. risk ratio, mean difference) used in the synthesis or presentation of results.	4-5
Synthesis methods	13a	Describe the processes used to decide which studies were eligible for each synthesis (e.g. tabulating the study intervention characteristics and comparing against the planned groups for each synthesis (item #5)).	4-5
	13b	Describe any methods required to prepare the data for presentation or synthesis, such as handling of missing summary statistics, or data conversions.	4-5
	13c	Describe any methods used to tabulate or visually display results of individual studies and syntheses.	4-5
	13d	Describe any methods used to synthesize results and provide a rationale for the choice(s). If meta-analysis was performed, describe the model(s), method(s) to identify the presence and extent of statistical heterogeneity, and software package(s) used.	4-5
	13e	Describe any methods used to explore possible causes of heterogeneity among study results (e.g. subgroup analysis, meta-regression).	4-5
	13f	Describe any sensitivity analyses conducted to assess robustness of the synthesized results.	4-5
Reporting bias assessment	14	Describe any methods used to assess risk of bias due to missing results in a synthesis (arising from reporting biases).	4-5
Certainty assessment	15	Describe any methods used to assess certainty (or confidence) in the body of evidence for an outcome.	NA
**RESULTS**			
Study selection	16a	Describe the results of the search and selection process, from the number of records identified in the search to the number of studies included in the review,	5-6

Section and Topic	Item #	Checklist item	Location where item is reported
		ideally using a flow diagram.	
	16b	Cite studies that might appear to meet the inclusion criteria, but which were excluded, and explain why they were excluded.	NA
Study characteristics	17	Cite each included study and present its characteristics.	7-9
Risk of bias in studies	18	Present assessments of risk of bias for each included study.	7
Results of individual studies	19	For all outcomes, present, for each study: (a) summary statistics for each group (where appropriate) and (b) an effect estimate and its precision (e.g. confidence/credible interval), ideally using structured tables or plots.	7-9
Results of syntheses	20a	For each synthesis, briefly summarise the characteristics and risk of bias among contributing studies.	7
	20b	Present results of all statistical syntheses conducted. If meta-analysis was done, present for each the summary estimate and its precision (e.g. confidence/credible interval) and measures of statistical heterogeneity. If comparing groups, describe the direction of the effect.	7, 9
	20c	Present results of all investigations of possible causes of heterogeneity among study results.	NA
	20d	Present results of all sensitivity analyses conducted to assess the robustness of the synthesized results.	7
Reporting biases	21	Present assessments of risk of bias due to missing results (arising from reporting biases) for each synthesis assessed.	NA
Certainty of evidence	22	Present assessments of certainty (or confidence) in the body of evidence for each outcome assessed.	NA
**DISCUSSION**			
Discussion	23a	Provide a general interpretation of the results in the context of other evidence.	10-11
	23b	Discuss any limitations of the evidence included in the review.	12

Section and Topic	Item #	Checklist item	Location where item is reported
	23c	Discuss any limitations of the review processes used.	12
	23d	Discuss implications of the results for practice, policy, and future research.	12
**OTHER INFORMATION**			
Registration and protocol	24a	Provide registration information for the review, including register name and registration number, or state that the review was not registered.	3
	24b	Indicate where the review protocol can be accessed, or state that a protocol was not prepared.	3
	24c	Describe and explain any amendments to information provided at registration or in the protocol.	NA
Support	25	Describe sources of financial or non-financial support for the review, and the role of the funders or sponsors in the review.	13
Competing interests	26	Declare any competing interests of review authors.	13
Availability of data, code and other materials	27	Report which of the following are publicly available and where they can be found: template data collection forms; data extracted from included studies; data used for all analyses; analytic code; any other materials used in the review.	13

From: Page MJ, McKenzie JE, Bossuyt PM, Boutron I, Hoffmann TC, Mulrow CD, et al. The PRISMA 2020 statement: an updated guideline for reporting systematic reviews. BMJ 2021;372:n71. doi: 10.1136/bmj.n71

Supplementary Table 3: Search Strategy

**PubMed**
#1 (Nephrotic Syndrome[MeSH Terms]) OR (Nephrotic Syndrome[Title/Abstract])
#2 (resistant[Title/Abstract]) OR (resistance[Title/Abstract])
#3 steroid[Title/Abstract]
#4 (((((((((predict*[Title/Abstract]) OR (risk[Title/Abstract])) OR (biomarker*[Title/Abstract])) OR ("c statistic"[Title/Abstract])) OR (discrimination[Title/Abstract])) OR (calibration[Title/Abstract])) OR (AUC[Title/Abstract])) OR ("area under the curve"[Title/Abstract])) OR ("area under the receiver operator characteristic curve"[Title/Abstract])) OR (decision*[Title/Abstract])
#5 #1 AND #2 AND #3
#6 #4 AND #5
**Embase**
#1 'nephrotic syndrome'/exp
#2 'nephrotic syndrome':ab,ti
#3 resistant:ab,ti OR resistance:ab,ti
#4'steroid':ab,ti
#5 (((((((((predict*[Title/Abstract]) OR (risk[Title/Abstract])) OR (biomarker*[Title/Abstract])) OR ("c statistic"[Title/Abstract])) OR (discrimination[Title/Abstract])) OR (calibration[Title/Abstract])) OR (AUC[Title/Abstract])) OR ("area under the curve"[Title/Abstract])) OR ("area under the receiver operator characteristic curve"[Title/Abstract])) OR (decision*[Title/Abstract])
#6 #1 OR #2
#7 #3 AND #4 AND #6
#8 #5 AND #7
**Scopus**
#1 (TITLE-ABS-KEY (nephrotic AND syndrome) AND TITLE-ABS-KEY (resistant OR resistance) AND TITLE-ABS-KEY (steroid))
#2 TITLE-ABS-KEY (predict* OR risk OR biomarker* OR "c statistic" OR discrimination OR

calibration OR auc OR "area under the curve" OR "area under the receiver operator characteristic curve" OR decision*)

#3 #1 AND #2 ((TITLE-ABS-KEY (nephrotic AND syndrome) AND TITLE-ABS-KEY (resistant OR resistance) AND TITLE-ABS-KEY (steroid))) AND (TITLE-ABS-KEY (predict* OR risk OR biomarker* OR "c statistic" OR discrimination OR calibration OR auc OR "area under the curve" OR "area under the receiver operator characteristic curve" OR decision*))

China National Knowledge Infrastructure: CNKI

(SU %='nephrotic syndrome' OR TKA =' nephrotic syndrome”) AND (TKA ='risk' OR TKA ='predict' OR TKA = 'biomarker') AND (TKA = 'resistant' OR TKA = 'resistance') AND (TKA = 'steroid')

Wan Fang

(Theme:(nephrotic syndrome) or Abstract:(nephrotic syndrome)) and (Abstract:(risk) or Abstract:(predict) or Abstract:(biomarker)) and (Abstract:(resistant) or Abstract:(resistance)) and (Abstract:(steroid))

VIP

(U= nephrotic syndrome OR M= nephrotic syndrome) AND M=steroid AND (M=resistant OR M=resistance) AND (M=predict OR M=risk OR M=biomarker)

Sino Med

"nephrotic syndrome"[Abstract: Intelligence] AND "steroid"[Abstract: Intelligence] AND("resistant"[Abstract: Intelligence] OR "resistance"[Abstract: Intelligence]) AND("predict"[Abstract: Intelligence] OR "risk"[Abstract: Intelligence] OR "biomarker"[Abstract: Intelligence])

Supplementary Table 4: Outcomes of studies reporting on prediction models

Study	Study Aim	EPV	Predictors
Agrawal 2021 [1]	D	4.7	Serum: interleukin -7, interleukin -9, monocyte chemoattractant protein 1
Agrawal 2020 [2]	D	4.3	Serum: vitamin D binding protein, adiponectin, matrix metalloproteinase 2
Bennett 2017 [3]-Model 1	D	2.0	Urine: vitamin D binding protein, prealbumin, neutrophil gelatinase–associated lipocalin, fetuin-A, α-1 acid glycoprotein 1, α-1 acid glycoprotein 2, α-2 macroglobulin, Hemopexin, thyroxine-binding globulin, α-1-B glycoprotein
Bennett 2017 [3]-Model 2	D	4.0	Urine: vitamin D binding protein, prealbumin, neutrophil gelatinase–associated lipocalin, fetuin-A, and α-1 acid glycoprotein 2
Burlaka 2022 [4]-Model 1	D	15.0	Serum: white blood cell, red blood cell
Burlaka 2022 [4]-Model 2	D	10.0	Serum: creatinine, urea, cholesterol
Gooding 2020 [5]-Model 1	D	7.0	Age<br>Serum: glutamine
Gooding 2020 [5]-Model 2	D	7.0	Age<br>Serum: creatinine
Hong 2022 [6]	D	20.5	Serum: white blood cell<br>Urine: retinol -binding protein
Jiang 2019 [7]	D	8.0	Urine: retinol -binding protein, N-acetyl-β-D-glucosaminidase
Kou 2023 [8]-Model 1	D	10.8	Serum: erythrocyte sedimentation rate, 25-hydroxyvitamin D, suppressor T cells, D-dimer, fibrin degradation products, β2-microglobulin
Kou 2023 [8]-Model 2	D	16.3	Serum: erythrocyte sedimentation rate, suppressor T cells, D-dimer, β2-microglobulin
Ling 2019 [9]	D	22.0	Serum: immunoglobulin E, immunoglobulin G
Liu 2023 [10]	D	15.5	Serum: endothelin-1, polyligandproteoglynan-1
Wang 2012 [11]	D	6.3	Serum: globulin<br>Urine: α1-antitrypsin/creatinine, αl -microglobulin

Ye 2023 [12]	D	4.3	Vinculin autoantibody<br>Serum: erythrocyte sedimentation rate, percentage of neutrophils, cholesterol, prolonged prothrombin time, immunoglobulin A, aspartate aminotransferase, aspartate aminotransferase<br>Urine: occult blood
Ye 2023 [12]	EV	2.5	Vinculin autoantibody<br>Serum: erythrocyte sedimentation rate, percentage of neutrophils, cholesterol, prolonged prothrombin time, immunoglobulin A, aspartate aminotransferase, aspartate aminotransferase<br>Urine: occult blood
Zhang 2015 [13]	D	12.0	Age (>6.5)<br>Urine: microscopic hematuria, 24-hour urine protein

Note: **D:** derivation; **EPV:** events per variable; **EV:** external validation

Supplementary Table 4 Continued

Study	Modelling method	Internal validation	Handling of missing data	Handling of continuous predictors
Agrawal 2021 [1]	Logistic regression model	None	No missing values	Continuous
Agrawal 2020 [2]	Logistic regression model	None	No missing values	Continuous
Bennett 2017 [3]-Model 1	Logistic regression model	None	No missing values	Continuous
Bennett 2017 [3]-Model 2	Logistic regression model	None	No missing values	Continuous
Burlaka 2022 [4]-Model 1	Logistic regression model	None	Not reported	Continuous
Burlaka 2022 [4]-Model 2	Logistic regression model	None	Not reported	Continuous
Gooding 2020 [5]-Model 1	Logistic regression model	None	Exclusion of patients	Continuous
Gooding 2020 [5]-Model 2	Logistic regression model	None	Exclusion of patients	Continuous
Hong 2022 [6]	Logistic regression model	None	Exclusion of patients	Continuous
Jiang 2019 [7]	Logistic regression model	None	Not reported	Continuous
Kou 2023 [8]-Model 1	Logistic regression model	None	Exclusion of patients	Continuous
Kou 2023 [8]-Model 2	Logistic regression model	Bootstrapping	Exclusion of patients	Continuous
Ling 2019 [9]	Logistic regression model	None	Not reported	Continuous
Liu 2023 [10]	Logistic regression model	None	Exclusion of patients	Categorical/dichotomous
Wang 2012 [11]	Logistic regression model	None	Not reported	Continuous
Ye 2023 [12]	Machine learning	Cross validation	Exclusion of patients	Continuous
Ye 2023 [12]-external validation	None	None	None	None
Zhang 2015 [13]	Logistic regression model	None	Not reported	Categorical/dichotomous

Supplementary Table 4 Continued

Study	Model discrimination		Model classification		Model calibration	Clinical usefulness	Model presentation
	AUC	95% CI	Sensitivity	Specificity			
Agrawal 2021 [1]	0.85	0.72-0.97	0.64	0.85	None	None	Full equation
Agrawal 2020 [2]	0.78	None	None	None	None	None	None
Bennett 2017 [3]-Model 1	0.92	0.85-0.99	0.80	0.87	None	None	Full equation
Bennett 2017 [3]-Model 2	0.85	0.74-0.96	0.70	0.87	None	None	Full equation
Burlaka 2022 [4]-Model 1	0.75	0.63-0.88	None	None	None	None	None
Burlaka 2022 [4]-Model 2	0.88	0.78-0.97	None	None	None	None	None
Gooding 2020 [5]-Model 1	0.81	None	None	None	Hosmer-Lemeshow test	None	None
Gooding 2020 [5]-Model 2	0.80	None	None	None	Hosmer-Lemeshow test	None	None
Hong 2022 [6]	0.91	0.87-0.96	0.66	0.96	None	None	Nomogram
Jiang 2019 [7]	0.96	None	0.94	0.83	None	None	Full equation
Kou 2023 [8]-Model 1	0.87	None	0.83	0.77	Hosmer-Lemeshow test, Calibration plot	None	None
Kou 2023 [8]-Model 2	0.84	None	0.87	0.68	Hosmer-Lemeshow test, Calibration plot, Brier score	Decision curve analysis	Nomogram
Ling 2019 [9]	0.94	0.90-0.97	0.90	0.96	None	None	None
Liu 2023 [10]	0.90	0.83-0.95	None	None	None	Decision curve analysis	None
Wang 2012 [11]	0.94	None	0.95	0.83	None	None	None
Ye 2023 [12]	0.95	None	0.87	0.93	None	None	Full equation, Web
Ye 2023 [12]-external validation	0.94*	None	0.90	0.97	None	None	Full equation, Web
Zhang 2015 [13]	0.86	None	0.57	0.97	None	None	Full equation

* The model performance is evaluated based on accuracy.

Supplementary Table 5: Risk of bias and applicability assessment for each PROBAST domain

Study	ROB Participants	ROB Predictors	ROB Outcome	ROB Analysis	Applicability Participants	Applicability Predictors	Applicability Outcomes	ROB Overall	Overall Applicability
Agrawal 2021 [1]	U	L	L	H	U	L	L	H	U
Agrawal 2020 [2]	U	L	L	H	U	L	L	H	U
Bennett 2017 [3]	L	L	L	H	L	L	L	H	L
Burlaka 2022 [4]	U	L	L	U	U	L	L	U	U
Gooding 2020 [5]	L	L	L	H	L	L	L	H	L
Hong 2022 [6]	L	L	L	H	L	L	L	H	L
Jiang 2019 [7]	L	L	L	U	L	L	L	U	L
Kou 2023 [8]	L	L	L	H	L	L	L	H	L
Ling 2019 [9]	L	L	L	H	L	L	L	H	L
Liu 2023 [10]	L	L	L	U	L	L	L	U	L
Wang 2012 [11]	U	L	L	H	U	L	L	H	U
Ye 2023 [12]	L	L	L	H	L	L	L	H	L
Zhang 2015 [13]	L	H	H	H	L	L	L	H	L

Note: **H:** High Risk of Bias; **L:** Low Risk of Bias; **U:** Unclear Risk of Bias

Supplementary Table 6: Details of PROBAST risk-of-bias assessment

PROBAST	Agrawal 2021 [1]	Agrawal 2020 [2]	Bennett 2017 [3]	Burlaka 2022 [4]	Gooding 2020 [5]	Hong 2022 [6]
**1 Participants**						
1.1 Were appropriate data sources used, e.g., cohort, RCT, or nested case-control study data?	PY	PY	PY	PY	PY	PY
1.2 Were all inclusions and exclusions of participants appropriate?	NI	NI	PY	NI	PY	PY
**2 Predictors**						
2.1 Were predictors defined and assessed in a similar way for all participants?	Y	Y	Y	Y	Y	PY
2.2 Were predictor assessments made without knowledge of outcome data?	Y	Y	Y	Y	Y	Y
2.3 Are all predictors available at the time the model is intended to be used?	Y	Y	Y	Y	Y	Y
**3 Outcome**						
3.1 Was the outcome determined appropriately?	Y	Y	Y	Y	Y	Y
3.2 Was a prespecified or standard outcome definition used?	Y	Y	Y	Y	Y	Y
3.3 Were predictors excluded from the outcome definition?	Y	Y	Y	Y	Y	Y
3.4 Was the outcome defined and determined in a similar way for all participants?	Y	Y	Y	Y	Y	Y
3.5 Was the outcome determined without knowledge of predictor information?	Y	Y	Y	Y	Y	Y
3.6 Was the time interval between predictor assessment and outcome determination appropriate?	PY	PY	PY	PY	PY	Y

**4 Analysis**						
4.1 Were there a reasonable number of participants with the outcome?	N	N	PN	PY	N	Y
4.2 Were continuous and categorical predictors handled appropriately?	PY	PY	PY	PY	PY	PY
4.3 Were all enrolled participants included in the analysis?	Y	Y	Y	Y	Y	Y
4.4 Were participants with missing data handled appropriately?	NI	NI	NI	NI	N	PN
4.5 Was selection of predictors based on univariable analysis avoided? (Development only)	Y	Y	Y	Y	Y	Y
4.6 Were complexities in the data (e.g., censoring, competing risks, sampling of control participants) accounted for appropriately?	NI	NI	PY	NI	PN	PY
4.7 Were relevant model performance measures evaluated appropriately?	NI	NI	NI	NI	PN	NI
4.8 Were model overfitting and optimism in model performance accounted for? (Development only)	NI	NI	NI	NI	NI	NI
4.9 Do predictors and their assigned weights in the final model correspond to the results from the reported multivariable analysis? (Development only)	Y	Y	Y	Y	Y	Y

Note: **Y:** Yes; **PY:** Probably Yes; **N:** No; **PN:** Probably No; **NI:** No Information

Supplementary Table 6 Continued

PROBAST	Jiang 2019 [7]	Kou 2023 [8]	Ling 2019 [9]	Liu 2023 [10]	Wang 2012 [11]	Ye 2023 [12]	Zhang 2015 [13]
**1 Participants**							
1.1 Were appropriate data sources used, e.g., cohort, RCT, or nested case-control study data?	PY	PY	PY	PY	PY	PY	PY
1.2 Were all inclusions and exclusions of participants appropriate?	PY	Y	PY	PY	NI	Y	PY
**2 Predictors**							
2.1 Were predictors defined and assessed in a similar way for all participants?	Y	PY	PY	Y	Y	Y	Y
2.2 Were predictor assessments made without knowledge of outcome data?	Y	Y	Y	Y	Y	Y	PN
2.3 Are all predictors available at the time the model is intended to be used?	Y	Y	Y	Y	Y	Y	Y
**3 Outcome**							
3.1 Was the outcome determined appropriately?	Y	Y	Y	Y	Y	Y	Y
3.2 Was a prespecified or standard outcome definition used?	Y	Y	Y	Y	Y	Y	Y
3.3 Were predictors excluded from the outcome definition?	Y	Y	Y	Y	Y	Y	PN
3.4 Was the outcome defined and determined in a similar way for all participants?	Y	Y	Y	Y	Y	Y	Y
3.5 Was the outcome determined without knowledge of predictor information?	Y	Y	Y	Y	Y	Y	PN
3.6 Was the time interval between predictor assessment and outcome determination appropriate?	Y	PY	Y	PY	Y	PY	PY

4 Analysis							
4.1 Were there a reasonable number of participants with the outcome?	Y	Y	Y	Y	N	N	Y
4.2 Were continuous and categorical predictors handled appropriately?	PY	PY	PY	PY	PY	PY	PN
4.3 Were all enrolled participants included in the analysis?	Y	Y	Y	Y	Y	Y	Y
4.4 Were participants with missing data handled appropriately?	NI	PN	PN	NI	NI	N	NI
4.5 Was selection of predictors based on univariable analysis avoided? (Development only)	Y	Y	Y	Y	Y	Y	Y
4.6 Were complexities in the data (e.g., censoring, competing risks, sampling of control participants) accounted for appropriately?	NI	PY	PY	NI	NI	PN	NI
4.7 Were relevant model performance measures evaluated appropriately?	NI	PN	NI	NI	NI	NI	NI
4.8 Were model overfitting and optimism in model performance accounted for? (Development only)	NI	Y	NI	NI	NI	PY	NI
4.9 Do predictors and their assigned weights in the final model correspond to the results from the reported multivariable analysis? (Development only)	Y	Y	Y	Y	Y	Y	Y

Note: **Y:** Yes; **PY:** Probably Yes; **N:** No; **PN:** Probably No; **NI:** No Information

Supplementary Table 7: Risk of bias and applicability assessment for each NOS domain

Study	Selection	Comparability	Outcome	NOS Score
Agrawal 2021 [1]	4	2	1	7
Agrawal 2020 [2]	4	2	1	7
Cicek 2024 [14]	2	2	1	5
Cuzzoni 2019 [15]	3	2	1	6
Gooding 2020 [5]	4	2	1	7
Hong 2022 [6]	3	2	1	6
Imbusi 2020 [16]	3	2	1	6
Kifle 2020 [17]	3	2	1	6
Konstantelos 2019 [18]	4	2	2	8
Kou 2023 [8]	4	2	1	7
Li 2024 [19]	4	2	1	7
Ling 2019 [9]	4	2	1	7
Liu 2023 [10]	4	2	1	7
Rehman 2022 [20]	4	2	1	7
Salah 2023 [21]	3	2	2	7
Udagawa 2021 [22]	4	2	1	7
Wang 2012 [11]	4	2	1	7
Yin 2010 [23]	4	2	1	7
Zhang 2015 [13]	4	2	1	7

Supplementary Figure 1: Forest plots for pooled analyses of risk factors associated with steroid-resistant nephrotic syndrome in children

Demographic/underlying disease

(1) Age

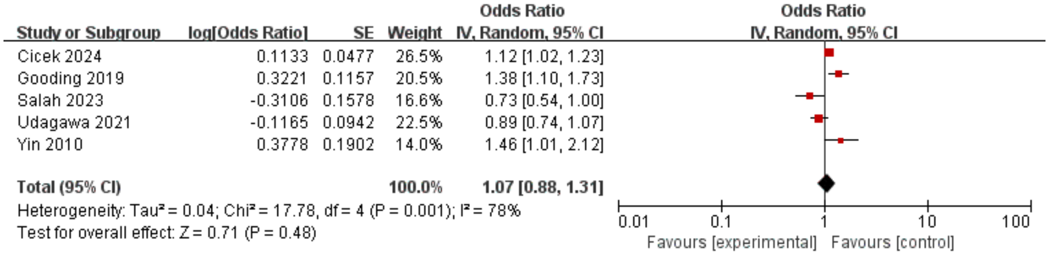

Definitions of age: Age was analyzed as a continuous variable, and the effect estimate corresponds to the change in SRNS risk per 1-year increase in age, as reported in the original studies.

(2) Age (> 4 years)

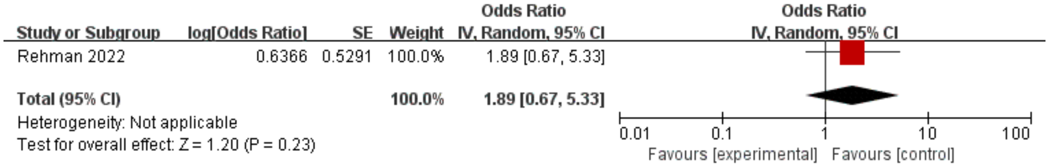

(3) Age (> 6 years)

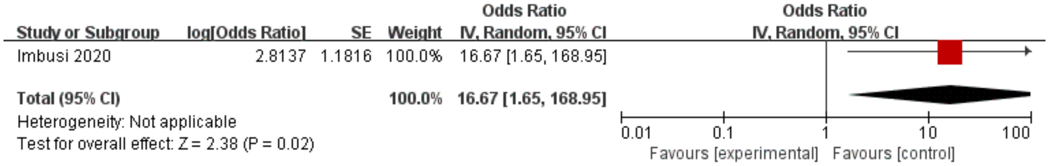

(4) Age (> 6.5 years)

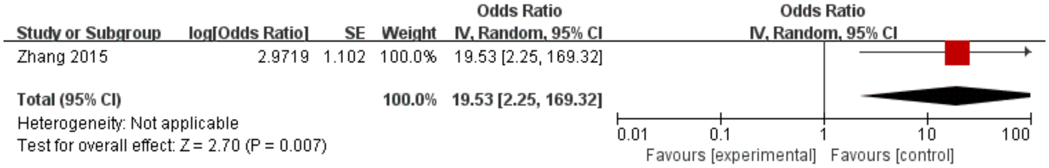

(5) Disease course

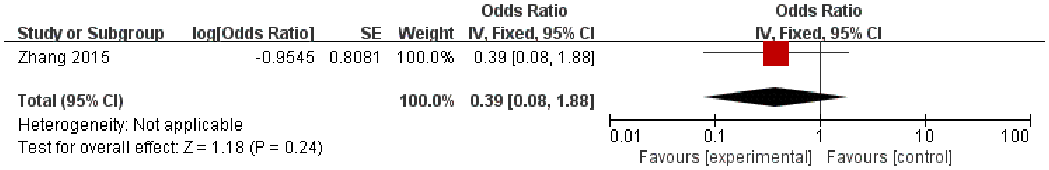

(6) Enlarged kidneys or Diffuse lesion

Study or Subgroup	log[]	SE	Weight	IV, Fixed, 95% CI
Zhang 2015	-0.5344	0.7605	100.0%	0.59 [0.13, 2.60]
**Total (95% CI)**			**100.0%**	**0.59 [0.13, 2.60]**

Heterogeneity: Not applicable
Test for overall effect: Z = 0.70 (P = 0.48)

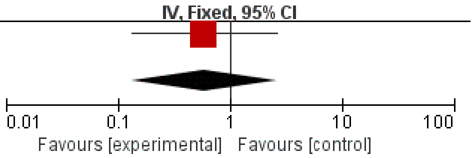

(7) Gender (Female=1)

Study or Subgroup	log[Odds Ratio]	SE	Weight	Odds Ratio IV, Random, 95% CI
Hong 2022	0.3988	0.5178	36.0%	1.49 [0.54, 4.11]
Rehman 2022	-0.5978	0.5423	35.4%	0.55 [0.19, 1.59]
Udagawa 2021	2.2354	0.8077	28.5%	9.35 [1.92, 45.53]
**Total (95% CI)**			**100.0%**	**1.77 [0.43, 7.34]**

Heterogeneity: $Tau^2 = 1.19$; $Chi^2 = 8.51$, df = 2 (P = 0.01); $I^2 = 76\%$
Test for overall effect: Z = 0.79 (P = 0.43)

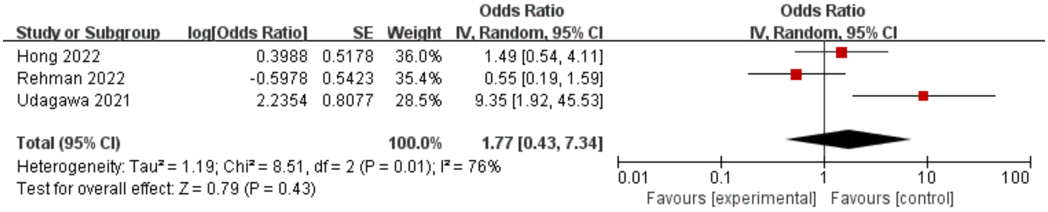

Definitions of gender: Gender was coded as female versus male. For Hong 2022, which originally coded male as the exposure, the effect estimate was inverted to ensure consistency with the other studies.

(8) Hypertension

Study or Subgroup	log[Odds Ratio]	SE	Weight	Odds Ratio IV, Random, 95% CI
Cicek 2024	0.6313	0.5913	41.4%	1.88 [0.59, 5.99]
Kifle 2020	1.9015	1.3447	16.8%	6.70 [0.48, 93.42]
Rehman 2022	2.3527	0.5836	41.8%	10.51 [3.35, 33.00]
**Total (95% CI)**			**100.0%**	**4.78 [1.35, 16.85]**

Heterogeneity: $Tau^2 = 0.65$; $Chi^2 = 4.37$, df = 2 (P = 0.11); $I^2 = 54\%$
Test for overall effect: Z = 2.43 (P = 0.02)

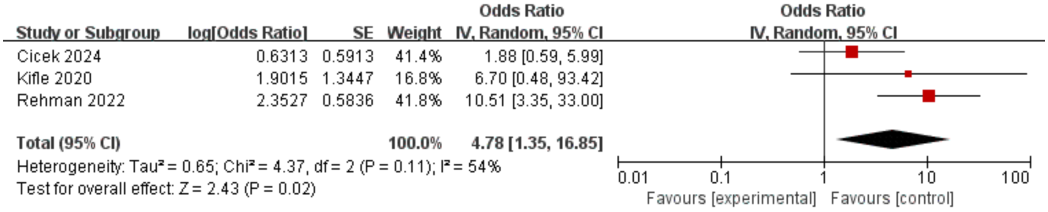

Definition of hypertension: Hypertension was defined as systolic or diastolic blood pressure above the 95th percentile for age, sex, and height at presentation.

(9) Low birth weight

Study or Subgroup	log[Odds Ratio]	SE	Weight	Odds Ratio IV, Fixed, 95% CI
Konstantelos 2019	1.3297	0.5525	100.0%	3.78 [1.28, 11.16]
**Total (95% CI)**			**100.0%**	**3.78 [1.28, 11.16]**

Heterogeneity: Not applicable
Test for overall effect: Z = 2.41 (P = 0.02)

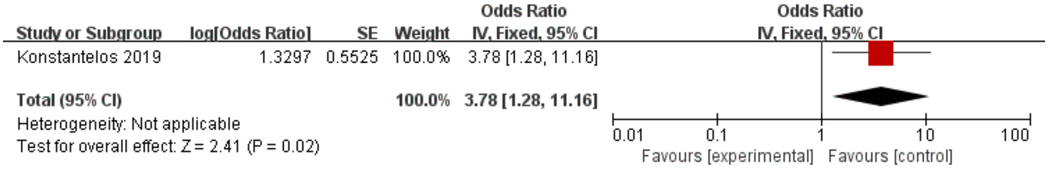

(10) Medication adherence

Study or Subgroup	log[Odds Ratio]	SE	Weight	Odds Ratio IV, Fixed, 95% CI
Kifle 2020	-1.7898	1.0828	100.0%	0.17 [0.02, 1.39]
**Total (95% CI)**			**100.0%**	**0.17 [0.02, 1.39]**

Heterogeneity: Not applicable
Test for overall effect: Z = 1.65 (P = 0.10)

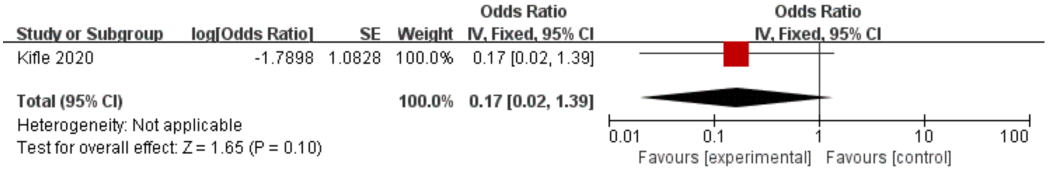

Biochemical indicators

Serum

(11) 25-hydroxyvitamin D

Study or Subgroup	log[Odds Ratio]	SE	Weight	Odds Ratio IV, Fixed, 95% CI
Kou 2023	-0.0305	0.0215	100.0%	0.97 [0.93, 1.01]
**Total (95% CI)**			**100.0%**	**0.97 [0.93, 1.01]**
Heterogeneity: Not applicable				
Test for overall effect: Z = 1.42 (P = 0.16)				

Odds Ratio IV, Fixed, 95% CI

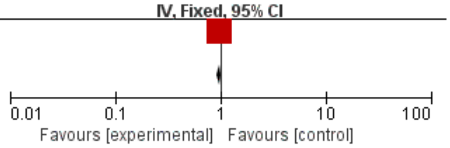

(12) Albumin

Study or Subgroup	log[Odds Ratio]	SE	Weight	Odds Ratio IV, Fixed, 95% CI
Yin 2010	0.1689	0.0696	100.0%	1.18 [1.03, 1.36]
**Total (95% CI)**			**100.0%**	**1.18 [1.03, 1.36]**
Heterogeneity: Not applicable				
Test for overall effect: Z = 2.43 (P = 0.02)				

Odds Ratio IV, Fixed, 95% CI

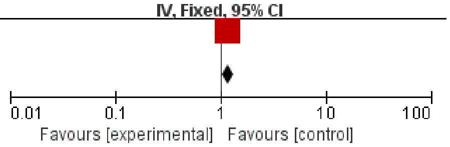

(13) Cholesterol

Study or Subgroup	log[Odds Ratio]	SE	Weight	Odds Ratio IV, Random, 95% CI
Imbusi 2020	0.0129	0.004	100.0%	1.01 [1.01, 1.02]
**Total (95% CI)**			**100.0%**	**1.01 [1.01, 1.02]**
Heterogeneity: Not applicable				
Test for overall effect: Z = 3.23 (P = 0.001)				

Odds Ratio IV, Random, 95% CI

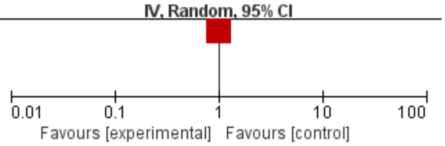

(14) Creatinine

Study or Subgroup	log[Odds Ratio]	SE	Weight	Odds Ratio IV, Fixed, 95% CI
Yin 2010	0	0.0278	100.0%	1.00 [0.95, 1.06]
**Total (95% CI)**			**100.0%**	**1.00 [0.95, 1.06]**
Heterogeneity: Not applicable				
Test for overall effect: Z = 0.00 (P = 1.00)				

Odds Ratio IV, Fixed, 95% CI

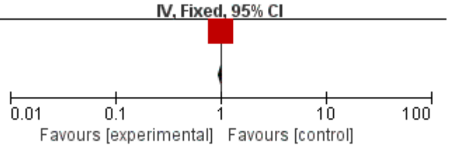

(15) D-dimer

Study or Subgroup	log[Odds Ratio]	SE	Weight	Odds Ratio IV, Random, 95% CI
Kou 2023	3.243	1.3218	52.6%	25.61 [1.92, 341.61]
Yin 2010	3.4099	1.3938	47.4%	30.26 [1.97, 464.84]
**Total (95% CI)**			**100.0%**	**27.72 [4.23, 181.60]**
Heterogeneity: Tau² = 0.00; Chi² = 0.01, df = 1 (P = 0.93); I² = 0%				
Test for overall effect: Z = 3.46 (P = 0.0005)				

Odds Ratio IV, Random, 95% CI

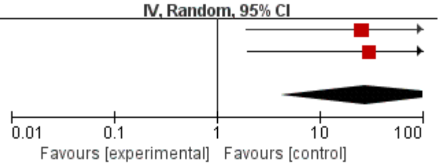

Definition of D-dimer: D-dimer was treated as a continuous variable, with the effect estimate corresponding to each 1 g/L increase in D-dimer level.

(16) Erythrocyte sedimentation rate

Study or Subgroup	log[Odds Ratio]	SE	Weight	Odds Ratio IV, Fixed, 95% CI
Kou 2023	0.0198	0.0101	100.0%	1.02 [1.00, 1.04]
**Total (95% CI)**			**100.0%**	**1.02 [1.00, 1.04]**
Heterogeneity: Not applicable				
Test for overall effect: Z = 1.96 (P = 0.05)				

Odds Ratio IV, Fixed, 95% CI

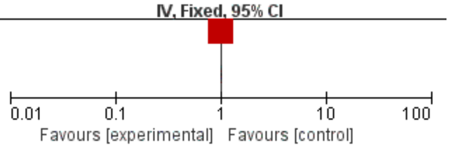

(17) Fibrinogen

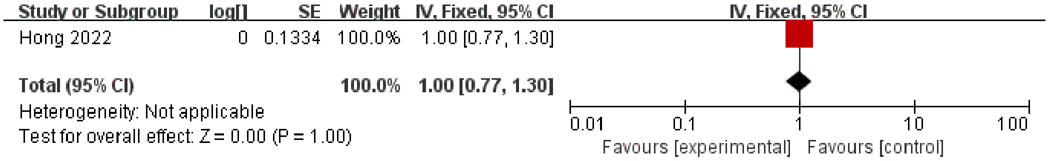

(18) Fibrin degradation products

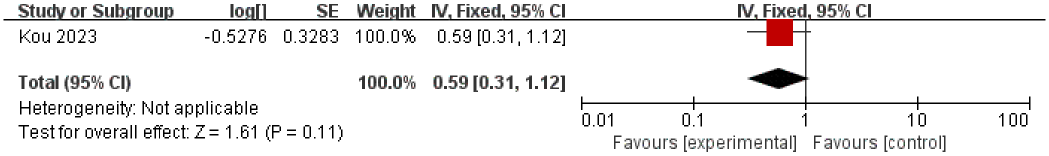

(19) Globulin

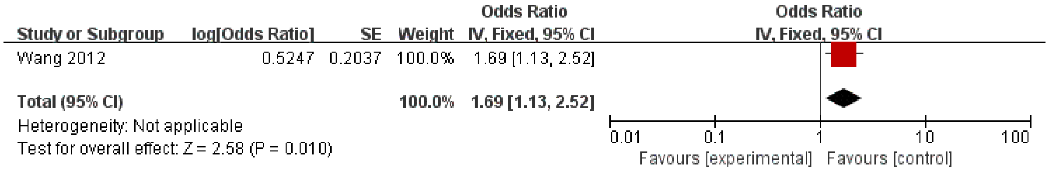

(20) Platelet/lymphocyte

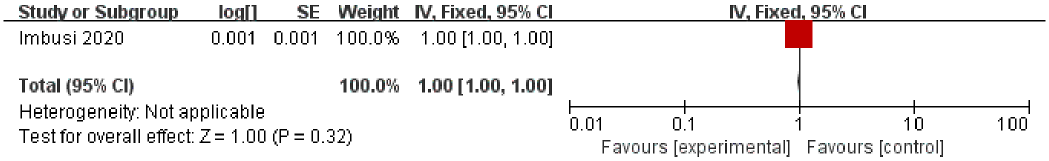

(21) Triglycerides

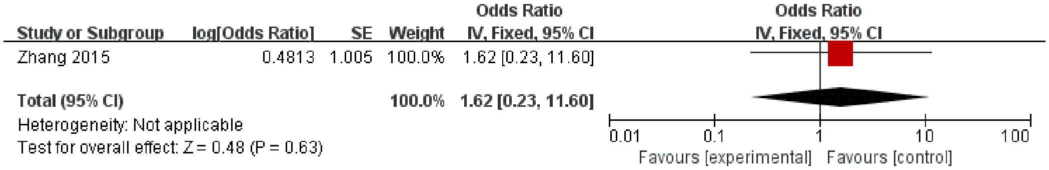

(22) White blood cells

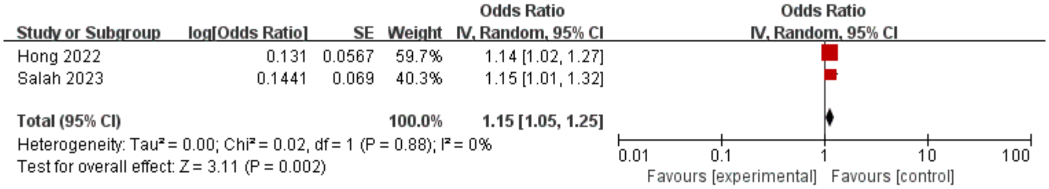

Definition of WBC: WBC was treated as a continuous variable, with the effect estimate corresponding to each 1×10^9/L increase in white blood cell count.

Urine

(23) Hematuria

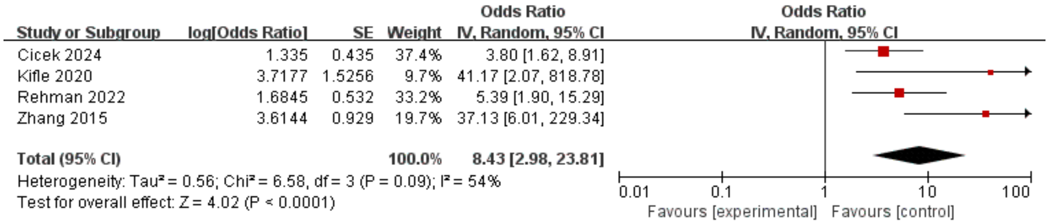

Definition of hematuria: Hematuria was defined as ≥5 or >5 red blood cells per high-power field in Kifle 2020 and Cicek 2024, ≥10 red blood cells per high-power field in Zhang 2015, and urine dipstick blood positivity of >+1 in Rehman 2022. Given that all definitions captured the presence of hematuria, these studies were pooled.

(24) Proteinuria

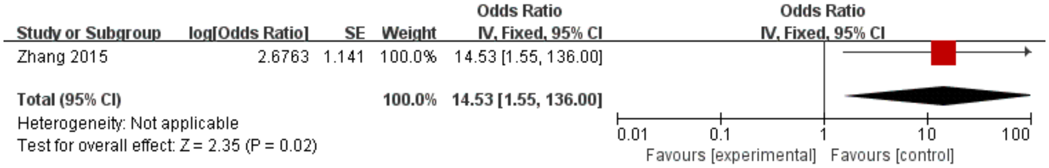

(25) Proteinuria/Creatinine (> 10g/g)

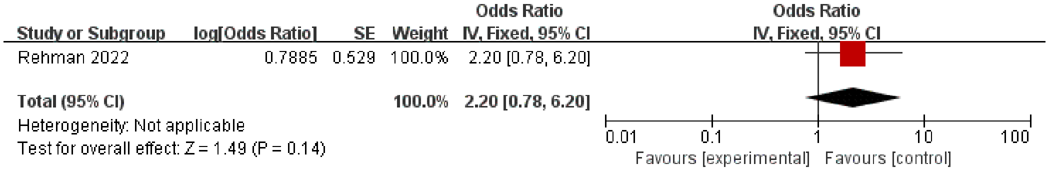

(26) Reduced urine output

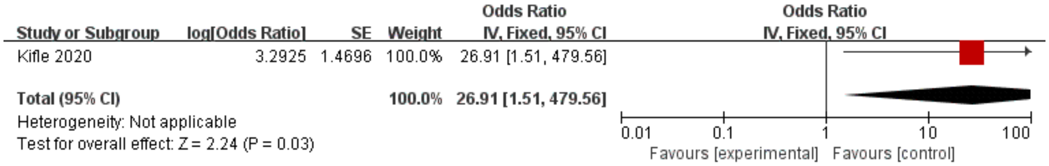

Immune/inflammatory factors

Serum

(27) CD3+ T cells

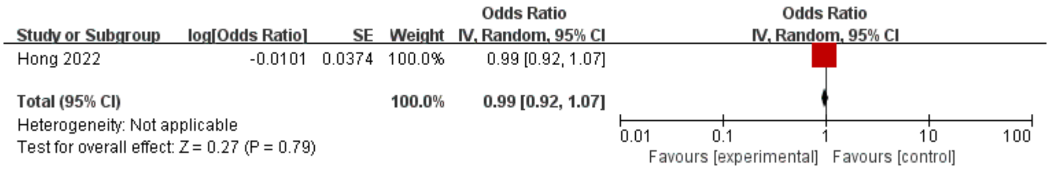

(28) CD3+CD4+T cells

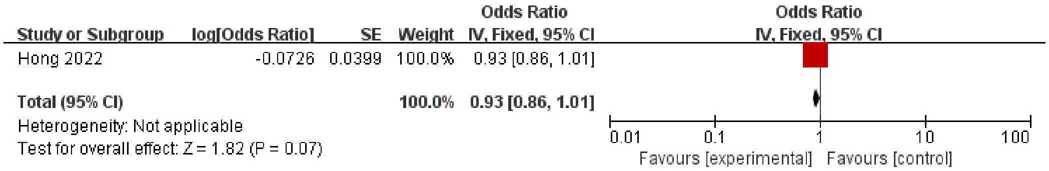

(29) CD4+/CD8+ T cells

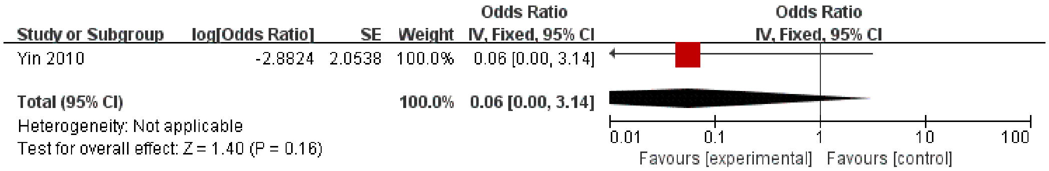

(30) CD8+ T cells

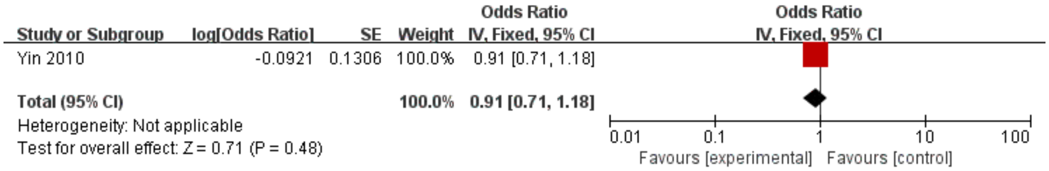

(31) Complement 3

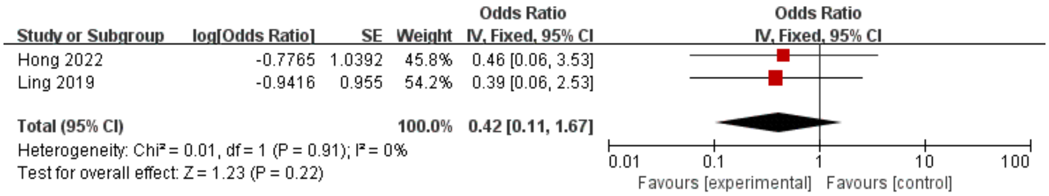

Definition of C3: Complement C3 was treated as a continuous variable, with the effect estimate corresponding to each 1 g/L increase in complement C3 level.

(32) Complement 4

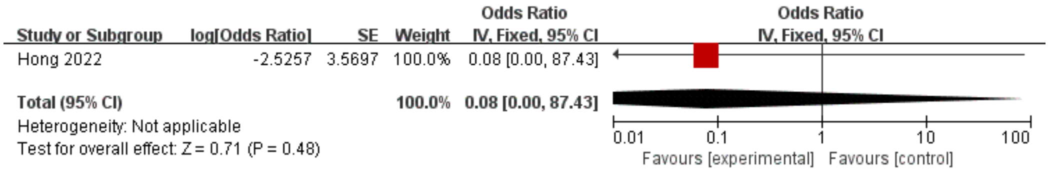

(33) Immunoglobulin E

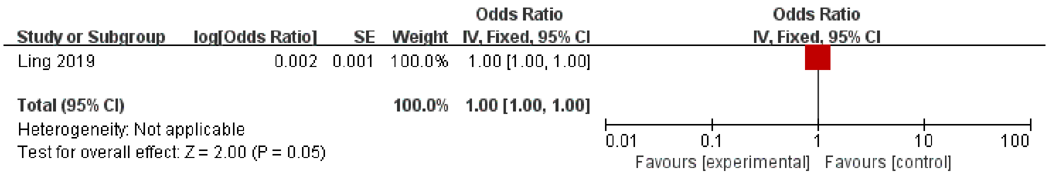

(34) Immunoglobulin G

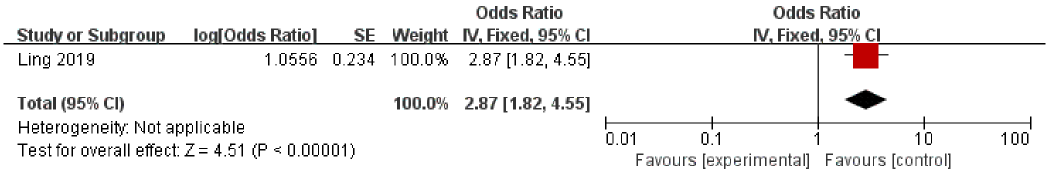

(35) Immunoglobulin M

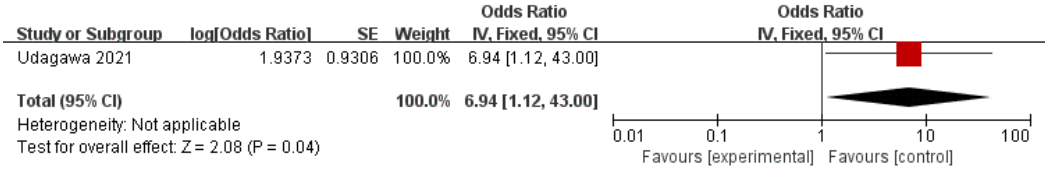

(36) Suppressor T cells

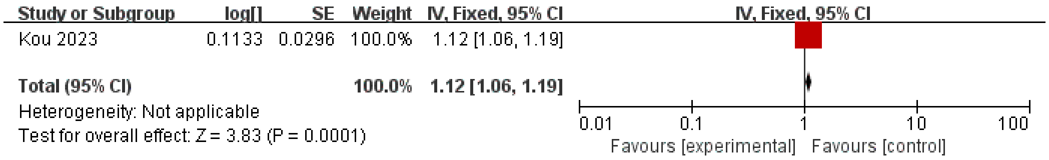

Metabolic biomarkers

Serum

(37) β1-globulin

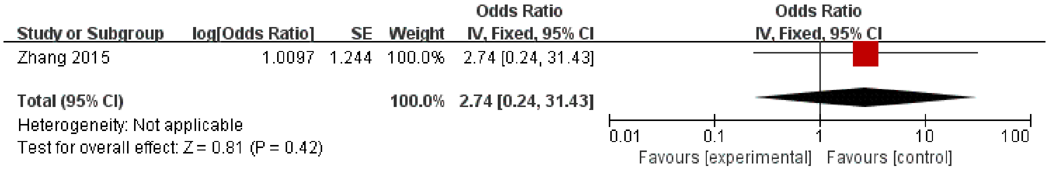

(38) β2-microglobulin

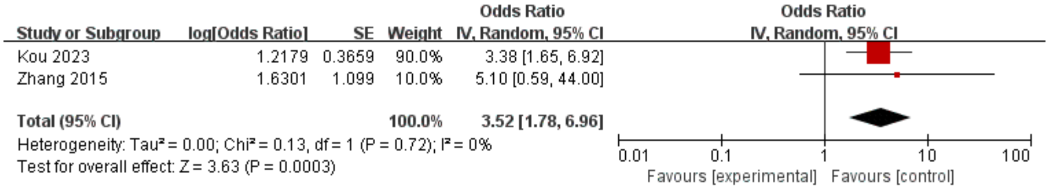

Definition of β2-microglobulin: β2-microglobulin was treated as a continuous variable, with the effect estimate corresponding to each 1 mg/L increase in β2-microglobulin level.

(39) Endothelin-1

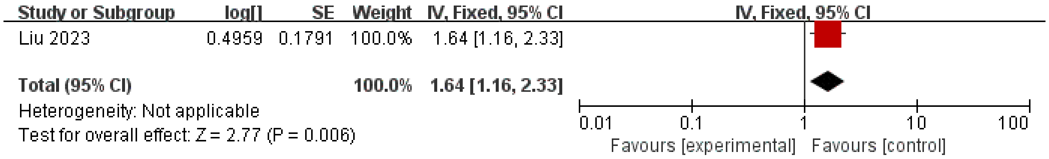

(40) Glutamine

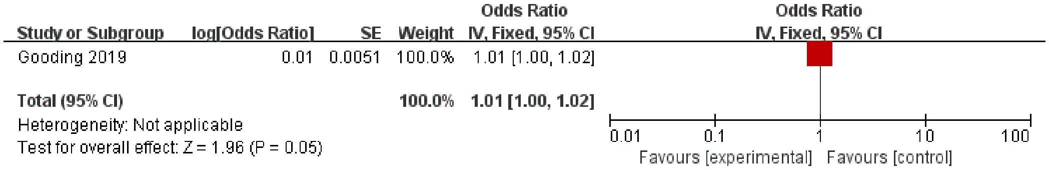

(41) Polyligand proteoglynan-1

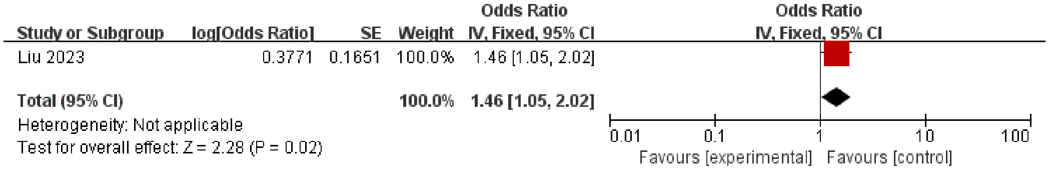

Urine

(42) α1-microglobulin

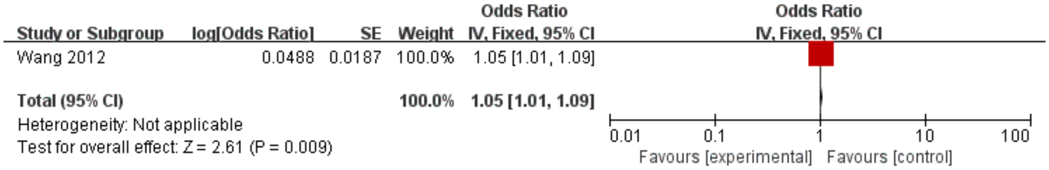

(43) α1-microglobulin/Creatine

Study or Subgroup	log[Odds Ratio]	SE	Weight	Odds Ratio IV, Fixed, 95% CI
Zhang 2015	0.424	1.039	100.0%	1.53 [0.20, 11.71]
**Total (95% CI)**			**100.0%**	**1.53 [0.20, 11.71]**
Heterogeneity: Not applicable				
Test for overall effect: Z = 0.41 (P = 0.68)				

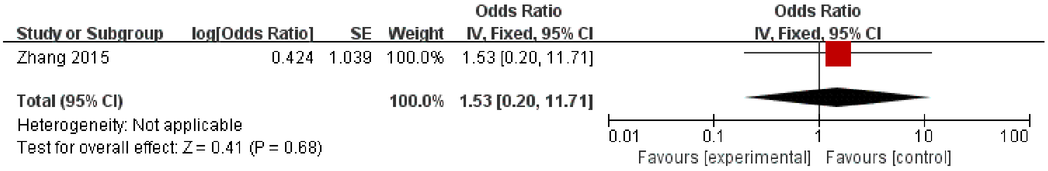

(44) β2-microglobulin

Study or Subgroup	log[Odds Ratio]	SE	Weight	Odds Ratio IV, Fixed, 95% CI
Rehman 2022	0.8329	0.5079	100.0%	2.30 [0.85, 6.22]
**Total (95% CI)**			**100.0%**	**2.30 [0.85, 6.22]**
Heterogeneity: Not applicable				
Test for overall effect: Z = 1.64 (P = 0.10)				

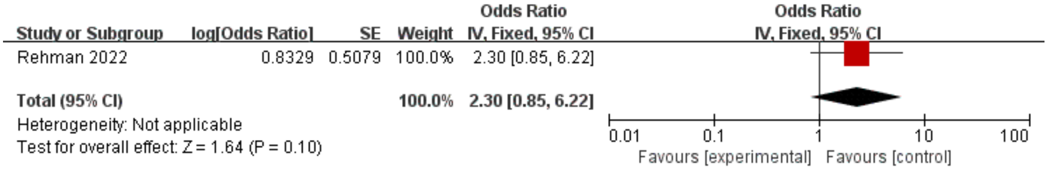

(45) β2-microglobulin/Creatine

Study or Subgroup	log[Odds Ratio]	SE	Weight	Odds Ratio IV, Fixed, 95% CI
Zhang 2015	1.3484	1.243	100.0%	3.85 [0.34, 44.02]
**Total (95% CI)**			**100.0%**	**3.85 [0.34, 44.02]**
Heterogeneity: Not applicable				
Test for overall effect: Z = 1.08 (P = 0.28)				

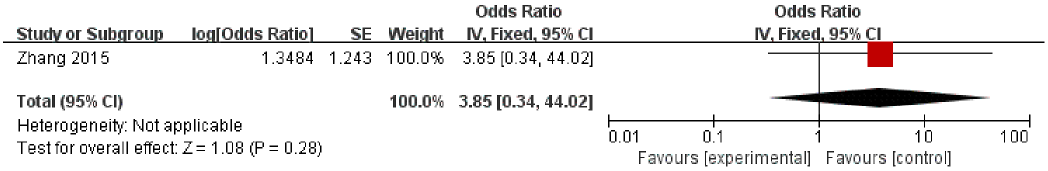

(46) Retinol binding protein

Study or Subgroup	log[Odds Ratio]	SE	Weight	Odds Ratio IV, Fixed, 95% CI
Hong 2022	0.0953	0.0486	100.0%	1.10 [1.00, 1.21]
**Total (95% CI)**			**100.0%**	**1.10 [1.00, 1.21]**
Heterogeneity: Not applicable				
Test for overall effect: Z = 1.96 (P = 0.05)				

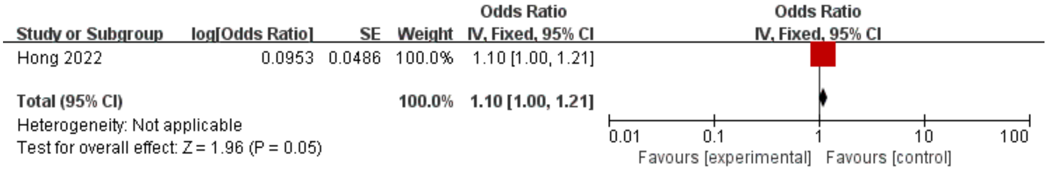

Reference:

1. Agrawal, S.; Brier, M.E.; Kerlin, B.A.; Smoyer, W.E. Plasma Cytokine Profiling to Predict Steroid Resistance in Pediatric Nephrotic Syndrome. *Kidney Int. Rep.* **2021**, *6*, 785–795.
2. Agrawal, S.; Merchant, M.L.; Kino, J.; Li, M.; Wilkey, D.W.; Gaweda, A.E.; Brier, M.E.; Chanley, M.A.; Gooding, J.R.; Sumner, S.J.; et al. Predicting and Defining Steroid Resistance in Pediatric Nephrotic Syndrome Using Plasma Proteomics. *Kidney Int. Rep.* **2020**, *5*, 66–80.
3. Bennett, M.R.; Pleasant, L.; Haffner, C.; Ma, Q.; Haffey, W.D.; Ying, J.; Wagner, M.; Greis, K.D.; Devarajan, P. A Novel Biomarker Panel to Identify Steroid Resistance in Childhood Idiopathic Nephrotic Syndrome. *Biomark. Insights* **2017**, *12*, 1177271917695832.
4. Burlaka, I.; Mityuryayeva, I.; Bagdasarova, I. Clinical and Apoptotic Factors Defining and Predicting Steroid Resistance in Nephrotic Syndrome in Children. *Glob. Pediatr. Health* **2022**, *9*, 2333794x221085392.
5. Gooding, J.R.; Agrawal, S.; McRitchie, S.; Acuff, Z.; Merchant, M.L.; Klein, J.B.; Smoyer, W.E.; Sumner, S.J. Predicting and Defining Steroid Resistance in Pediatric Nephrotic Syndrome Using Plasma Metabolomics. *Kidney Int. Rep.* **2020**, *5*, 81–93.
6. Hong, M.Z.; Zhong, P.Q.; Chen, M.Y.; Chen, P.S. Laboratory indicators predict the efficacy of glucocorticoid therapy in pediatric primary nephrotic syndrome. *J. Trop. Med.* **2022**, *22*, 1082–1087.
7. Jiang, Y.; Zhang, B.L.; Wang, W.H. Clinical significance of detection of urine renal injury biomarkers in children with primary nephrotic syndrome. *Chin. J. Appl. Clin. Pediatr.* **2019**, *34*, 1326–1330.
8. Kou, M.; Wu, F.; Qu, X.Y.; Wang, H.; Guo, X.T.; Yang, Y.Y.; Zhao, L.J. Establishment and validation of clinical prediction model for steroid-resistant nephrotic syndrome in children. *Chin. J. Pediatr.* **2023**, *61*, 333–338.
9. Ling, C.; Chen, Z.; Fan, J.F.; Sun, Q.; Meng, Q.; Hua, L.; Liu, X.R. Value of serum lgG combined with IgE in predicting steroid therapy response in children with primary nephrotic syndrome. *Chin. J. Nephrol.* **2019**, *35*, 835–840.
10. Liu, Q.; Zhu, J.; Tang, G.Y.; Jiang, P.Y. Serum levels of ET-1 and SDC-1 in children with nephrotic syndrome and their correlation with hormone therapy response. *Tianjin Med. J.* **2023**, *51*, 413–417.
11. Wang, Y.Y.; DIng, G.X.; Yuan, Y.G.; Bao, H.Y.; Chen, Y.; Zhao, F.; Han, Y.; Zhang, A.H.; Huang, S.M. Detection of urinary α1-antitrypsin for predicting response to glucocorticoid therapy in children with primary nephrotic syndrome. *Chin. J. Nephrol.* **2012**, *28*, 857–862.
12. Ye, Q.; Li, Y.; Liu, H.; Mao, J.; Jiang, H. Machine learning models for predicting steroid-resistant of nephrotic syndrome. *Front. Immunol.* **2023**, *14*, 1090241.
13. Zhang, B.L.; Liu, T.; Lin, S.X.; Wang, W.H.; Liu, Y.; Liu, Y.; Wu, X.; Wang, X.; Liu, Z. Analysis of risk factors for steroid resistance in children with primary nephritic syndrome and discussion on the prediction model of steroid resistance. *Chin. J. Nephrol.* **2015**, *31*, 414–418.
14. Cicek, N.; Yıldız, N.; Guven, S.; Kaya, M.; Gokce, I.; Alpay, H. Clinical Predictors of Steroid Resistance in Childhood Nephrotic Syndrome. *Clin. Pediatr.* **2024**, *63*, 1300–1307.
15. Cuzzoni, E.; Franca, R.; De Iudicibus, S.; Marcuzzi, A.; Lucafò, M.; Pelin, M.; Favretto, D.; Monti, E.; Morello, W.; Ghio, L.; et al. MIF plasma level as a possible tool to predict steroid responsiveness in children with idiopathic nephrotic syndrome. *Eur. J. Clin. Pharmacol.* **2019**, *75*, 1675–1683.
16. Imbusi, E.A.; Ekanem, P.E.; Gebrearegay, H.; Ambaye, M.; Tesfahunegn, A.; Nyaga, K.; Ekanem, R.; Peter, N. Steroid response pattern among children with nephrotic syndrome in Northern Ethiopia. *Nephro-Urol. Mon.* **202**0, *12*, e106995.
17. Kifle, M.; Shimelis, D. Predictors of resistance to steroids in pediatric nephrotic syndrome at a tertiary hospital, Addis Ababa. *Ethiop. J. Pediatr. Child Health* **2020**, *15, 5–15.*
18. Konstantelos, N.; Banh, T.; Patel, V.; Vasilevska-Ristovska, J.; Borges, K.; Hussain-Shamsy, N.; Noone, D.; Hebert, D.; Radhakrishnan, S.; Licht, C.P.B.; et al. Association of low birth weight and prematurity with clinical outcomes of childhood nephrotic syndrome: A prospective cohort study. *Pediatr. Nephrol.* **2019**, *34*, 1599–1605.

19. Li, J.Z.; Wang, Y. Influencing factors and hormone resistance of 510 children with nephrotic syndrome. *J. Public Health Prev. Med.* **2024**, *35*, 79–82.
20. Rehman, M.; Ali, A.; Ehsan, A.; Aziz, M.; Khatri, S.; Hashmi, S. Can Steroid Response in Idiopathic Childhood Nephrotic Syndrome be Predicted? A Single Center Quasi-Experimental Study. *Pak. Armed Forces Med. J.* **2022**, *72*, 980.
21. Salah, D.M.; Aoun, A.H.; Fahmy, B.S.; Zeid, A.; Fahmy, Y.A. Does Urinary Vitamin D-Binding Protein Have a Role in the Prediction of Steroid Resistance in Nephrotic Syndrome? A Cohort Study on Egyptian Children. *J. Compr. Pediatr.* **2023**, *14*, e130133.
22. Udagawa T, Matsuyama Y, Okutsu M, Motoyoshi Y, Okada M, Tada N, Kikuchi E, Shimoda M, Kanamori T, Omori T et al: Association between Immunoglobulin M and Steroid Resistance in Children with Nephrotic Syndrome: A Retrospective Multicenter Study in Japan. Kidney360 2021, 2, 487–493.
23. Yin, L.; Zhou, W.; Sun, H.; Jin, Y.L.; Zhou, Z.Y. Risk Factors of Steroid Resistance in Children with Primary Nephrotic Syndrome. *Chin. J. Appl. Clin. Pediatr.* **2010**, *25*, 1330–1332,1356.
